# Killing behaviors in rodents: from predation to infanticide

**DOI:** 10.3389/fnbeh.2026.1778618

**Published:** 2026-08-17

**Authors:** Raffaele d'Isa, Piotr Bebas, Michael H. Parsons, Korneliusz Kurek, Rafal Stryjek

**Affiliations:** 1 Institute of Experimental Neuroscience, IRCCS San Raffaele Scientific Institute, Milan, Italy; 2 Department of Animal Physiology, Institute of Experimental Zoology, Faculty of Biology, University of Warsaw, Warsaw, Poland; 3 Effective Urban Rodent Ecology Knowledge Alliance (EUREKA), Centre for Urban Ecological Solutions (CUES), Houston, TX, United States; 4 Masurian Centre for Biodiversity Research and Education, Faculty of Biology, University of Warsaw, Urwitałt, Mikołajki, Poland; 5 Institute of Psychology, Polish Academy of Sciences, Warsaw, Poland

**Keywords:** antipredatory behavior, infanticide, interspecific killing, intraspecific killing, lethal attacking, predation, rodents, social competition

## Abstract

Killing is the most extreme behavior that an animal can perform toward another. Rodents are one of the best examples of evolutionary adaptation, being the mammals with the highest number of species and having developed an impressive variety of forms and behaviors, which allow them to live and thrive in the most disparate habitats across all continents except for Antarctica. Moreover, rodents are the most widely employed animal model in biological research. An appropriate knowledge of the ethology of rodents is of particular importance both for basic science and for the employment of these animals as animal models. Since they are common targets of both terrestrial and aerial predators, rodents are frequently viewed merely as prey animals. Nevertheless, it is less known that rodents can also be proficient killers. In fact, most rodents are opportunistic predators. Additionally, rodents perform several non-predatory killing behaviors. However, due to historical reasons (according to the early traditional views, rodents were considered small, exclusively herbivorous animals lacking killing instincts), the killing behaviors of rodents have long been overlooked. In this article, we present an overview of rodent killing behaviors, considering a wide variety of species and genera. Killing behaviors are classified into four major categories: predation (upon invertebrates, fish, amphibians, reptiles, birds and mammals), antipredatory killing, social competition-induced killing (intraspecific and interspecific), and infanticide (non-parental and parental). Notably, killing behaviors are present in the majority of rodent species and can be found in all five rodent suborders (Myomorpha, Sciuromorpha, Hystricomorpha, Castorimorpha, and Anomaluromorpha). The present work is the first in which these diverse types of rodent killing behaviors are described together. This not only allows a synoptical view of killing behaviors, but can also aid in identifying the boundaries between the different categories, making the classification of the specific killing behaviors easier. In the final section, we discuss ethical issues related to research on rodent killing behaviors, and we propose methodological options for addressing such issues in future investigations of these behaviors.

## 1 Introduction

Killing is the most extreme behavior that an animal can perform toward another. Rodents are the most widely employed animal model in biological research (Makowska and Weary, 2021; Domínguez-Oliva et al., 2023). Moreover, they are the greatest example of mammalian evolutionary adaptation. Indeed, Rodentia is the mammalian order with the greatest number of species (over 40% of all mammals are rodents), and its species have developed an impressive variety of forms and behaviors, which allow them to live and thrive in the most disparate habitats across all continents except for Antarctica (Kay and Hoekstra, 2008). A proper knowledge of the behavior of rodents is of primary importance both for basic science and for the adoption of rodents as animal models.

Since they are frequently targeted by both terrestrial and aerial predators, rodents are commonly classified as prey animals. Nevertheless, it is less known that rodents can also be proficient killers. In fact, most rodents are opportunistic predators. However, due to historical reasons, the killing instincts of rodents have long been overlooked.

Traditionally, it was believed that rodents were strictly herbivorous and hence incapable of predating other animals. For example, the British naturalist George Robert Waterhouse (1810–1888), from the Natural History Museum in London, lapidarily stated: “They feed upon vegetable substances and are of small size” (Waterhouse, 1848). The French naturalist Paul Gervais (1816–1879), Professor of Zoology at the University of Montpellier, affirmed that they are “mostly herbivorous or frugivorous, sometimes omnivorous”^1^ (Gervais, 1854). The British zoologists William Flower^2^ (1831–1899) and Richard Lydekker (1849–1915), in their prominent handbook of mammalogy, stated with great assertiveness: “All the rodents are exclusively herbivorous and the whole of them gather their food by gnawing” (Flower and Lydekker, 1891).

In the traditional view, rodents were small, herbivorous prey animals lacking killing instincts^3^. However, between the 1950s and the present, a growing amount of evidence has pointed out that most rodents not only supplement their herbivorous diet by scavenging animal remains, but are also endowed with predatory killing instincts. In 1970, Stuart Omer Landry Jr. (1924–2015), Professor of Biology at the State University of New York at Binghamton, published the seminal and field-challenging article *The Rodentia as Omnivores*, in which he proposed a radical shift of view regarding rodent diet, compiling a staggering list of rodent species that have been documented to feed upon a wide variety of invertebrate and vertebrate animals. Nevertheless, while at present a rich collection of cases is available in the scientific literature, the predatory behaviors of rodents, as well as most of their other killing behaviors, are still not widely known among the scientific community.

**Figure 1:**
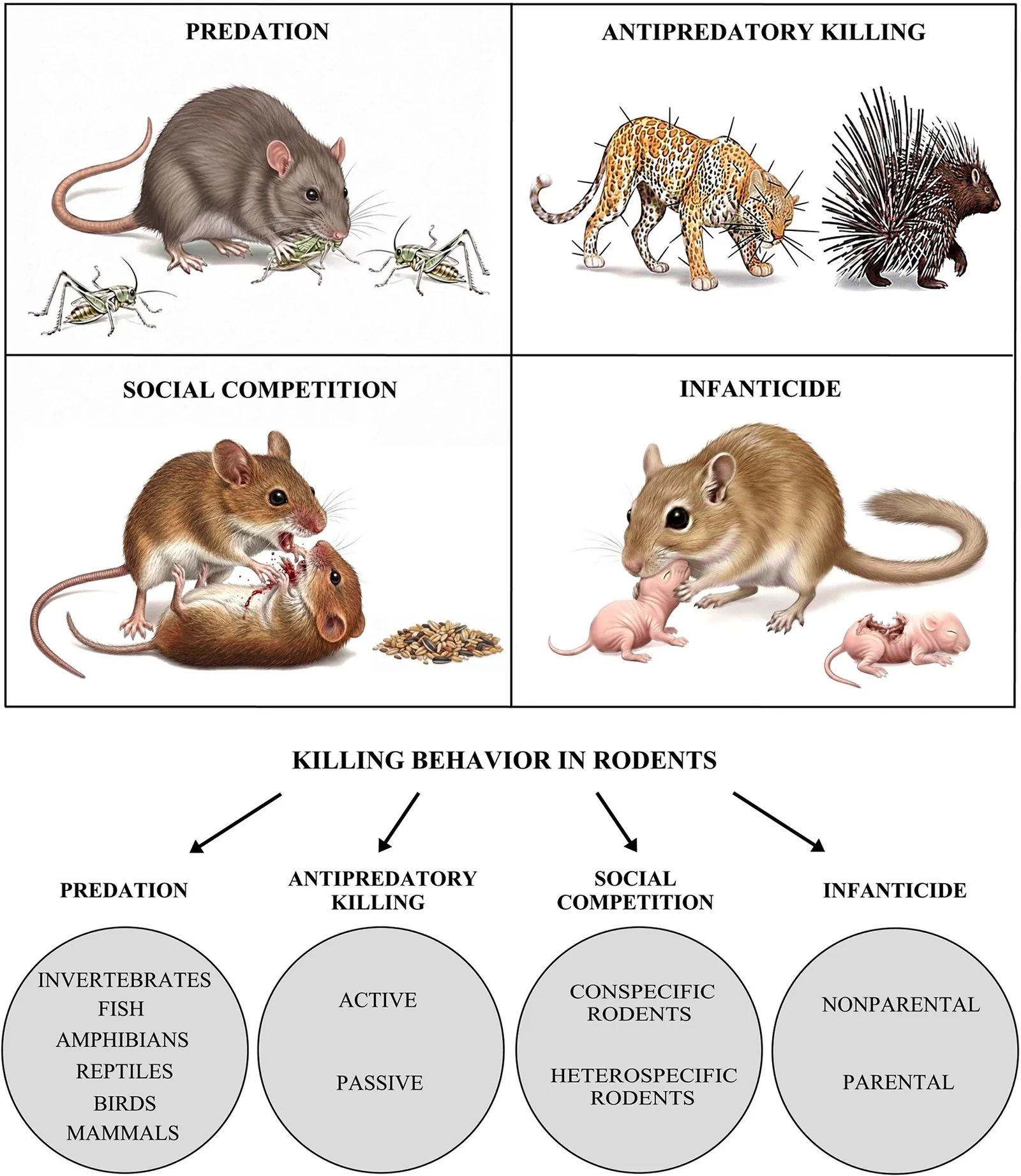
The four major categories of killing behavior in rodents. Predation: a Norway rat (*Rattus norvegicus*) predating on a cricket. Antipredatory killing: a crested porcupine (*Hystrix cristata*) lethally wounding a leopard (*Panthera pardus*). Social competition: a yellow-necked mouse (*Apodemus flavicollis*) killing a bank vole (*Clethrionomys glareolus*). Infanticide: a Mongolian gerbil (*Meriones unguiculatus*) killing a conspecific pup. Image created by Rafal Stryjek and Raffaele d'Isa.

In the present article, we will provide an overview of killing behaviors among rodents, describing both invertebrate- and vertebrate-killing behaviors. Importantly, rodents display both predatory and non-predatory killing behaviors. We here present a classification of rodent killing behaviors into four main categories: predation, antipredatory killing, intraspecific and interspecific social competition-induced killing, and infanticide. Figure 1 visualizes the classification of the main forms of rodent killing behaviors with their respective subtypes, and summarizes the structure that we will follow in the present review to describe killing behaviors. Remarkably, this is the first time that the different types of rodent killing behaviors are described together in a single review. Such an approach allows a more comprehensive and synoptical view of the killing behaviors. Moreover, considering these categories together can aid, by contrast, in the identification of the boundaries between the different categories. A better understanding of what distinguishes one category from another can facilitate the classification of the single killing behaviors, especially in the cases that may at first appear more difficult to interpret.

Embracing a comparative approach (d'Isa and Abramson, 2023), we will consider killing behaviors in a wide variety of rodent species, from the most diverse genera, showing how killing behaviors are present in all five rodent suborders (Myomorpha, Sciuromorpha, Hystricomorpha, Castorimorpha and Anomaluromorpha). Finally, in the conclusion, we will discuss ethical issues related to research on rodent killing behaviors, and we will address such issues by suggesting a set of methodological options for the future investigation of these behaviors.

## 2 Predatory killing

### 2.1 Invertebrate-killing by rodents

While traditionally considered just prey animals, rodents are actually predators as well. In fact, most rodents are, to one degree or another, predators of insects. For example, the house mouse (*Mus musculus*), when exposed to a cricket, promptly attacks and kills it (d'Isa, 2025a). From the 1960s, starting with the pioneering work of the comparative psychologist Karla Thomas Butler, this cricket-killing behavior of house mice has been widely studied in the laboratory, to the point that the cricket-killing test has become the main test of predatory behavior for this rodent species (Thomas, 1969; Butler, 1973; Lowe and O'Boyle, 1976; Yanai and Ginsburg, 1976; Whelton and O'Boyle, 1977; Nesbitt et al., 1979; Klunder and O'Boyle, 1979; Kantak et al., 1980; Hahn et al., 1991; Sandnabba, 1995; Gammie et al., 2003; Vishnivetskaya et al., 2007; Cai et al., 2020; Galvin et al., 2021; Johnson et al., 2021; Groves Kuhnle et al., 2022). Notably, mice's swift killing behavior in response to crickets is observed even if the mice never had any previous encounter with a cricket in their entire life, suggesting that cricket-killing is a highly instinctual response, and that both the motivation to kill and the skill to kill crickets are innate. Impressively, cricket-naïve mice captured crickets with an average latency of about 400 s from cricket presentation (Galvin et al., 2021). Nevertheless, learning can further increase the efficiency of cricket-killing. Indeed, when the same mice were newly exposed to crickets over the course of multiple days, a sharp decrease was observed in the time required for capture, which, by the eighth day, had dropped to less than 40 s (Galvin et al., 2021). Consumption time also decreased, with the mice becoming more skilled at handling and rotating the cricket for head removal (Galvin et al., 2021). Importantly, not all rodent killing behaviors are instinctual. Nevertheless, among all killing behaviors, predatory killing is the one that can be more clearly recognized as instinctual. The highly adaptive value of predatory hunting can easily explain the reason for which evolution has selected this behavior as an innate response in so many rodent species.

The heritability of the insect-killing behavior has been well demonstrated through experiments of artificial selection with bank voles (*Clethrionomys glareolus*) captured from Niepołomice Forest in southern Poland and then bred in the laboratory (Konczal et al., 2016). Basically, wild-caught bank voles were exposed to a cricket-killing test and then selected for low latency to capture the cricket. In unselected lines of bank voles, cricket-attacking behavior was naturally present but at a very low frequency: only about 20% of the voles attacked the insect. In contrast, after just 13 generations of selection, the cricket-attacking frequency of the voles stunningly increased to about 70%. Remarkably, this percentage was so high notwithstanding the fact that the tested voles had never encountered a cricket before, strongly suggesting an innate response. Analogously, the latency to attack was markedly decreased in the voles that had undergone selection. Four independent lines were selected for cricket-attacking and all four selection experiments led to the same outcome.

Grasshopper mice (a group of mouse species from the genus *Onychomys*) even take their name from their habit of predating on grasshoppers, which they hunt and kill in addition to other insects and non-insect arthropods, such as arachnids (mainly scorpions, to the venom of which they are resistant) and myriapods (including venomous centipedes) (Baxter, 1979; Langley and Knapp, 1982; Kemble and Lewis, 1982; Davies and Kemble, 1983; Langley, 1978, 1981a,b, 1983, 1986a,b, 1987, 1994, 1989, 1991, 2008; Rowe and Rowe, 2006; Rowe et al., 2013; Rowe and Rowe, 2015; Phillips et al., 2015; Shupe, 2024).

Zhanna Reznikova and colleagues have extensively investigated rodent hunting behavior in the laboratory using cockroaches as targets, documenting insect-killing behavior in a wide variety of rodent species, namely the black-striped mouse^4^ (*Apodemus agrarius*), the Norway rat (*Rattus norvegicus*), the Mongolian gerbil (*Meriones unguiculatus*), the fat-tailed gerbil (*Pachyuromys duprasi*), the East European vole (*Microtus levis*), the narrow-headed vole (*Lasiopodomys gregalis*), the Djungarian hamster^5^ (*Phodopus sungorus*), Campbell's dwarf hamster (*Phodopus campbelli*), the desert hamster (*Phodopus roborovskii*), the Mongolian hamster (*Allocricetulus curtatus*), Eversmann's hamster (*Allocricetulus eversmanni*), the Tuva silver vole (*Alticola tuvinicus*), the Olkhon mountain vole (*Alticola olchonensis*) and the flat-headed vole (*Alticola strelzowi*), as well as in a rodent-like non-rodent species, the common shrew (*Sorex araneus*), which from a comparative perspective can be interesting to study alongside rodents to understand convergent evolution and differences in killing behaviors among species adapted to similar ecological niches (Reznikova et al., 2017, 2019, 2022; Panteleeva et al., 2020; Levenets et al., 2024). Interestingly, the specific behavioral sequence employed for killing may vary across rodent species. The same group, in a comparative laboratory study of the insect-killing behavior of nine species (Norway rats, black-striped mice, Campbell's dwarf hamsters, Djungarian hamsters, Eversmann's hamsters, Mongolian hamsters, narrow-headed voles, Tuva silver voles and common shrews) examined, for each species, the alphabet of the hunting behavior, meaning the collection of the “letters” representing the single behavioral units of the hunting ethogram (Reznikova et al., 2019). This alphabet was composed of single behavioral units such as sniffing, pursuing the prey by walking, pursuing the prey by running, biting, carrying it in the teeth, seizing the insect with the forepaws, handling it, pinning it down to the ground with one or two paws, and nibbling the insect's legs. The team found that, while a set of core behavioral units was shared across all nine species, some species displayed notable differences. For example, the common shrew never seized the prey with its paws but only with its teeth. Moreover, the pinning down behavior (with one or two paws) was performed only by the shrews. The fact that the common shrews differed so markedly from all the other tested species is unsurprising, considering that they represented the only non-rodent species. Regarding the rodents, all four species of hamsters were capable of starting the attacks by seizing the cockroach with their paws, and did so in 20–25% of the cases. In contrast, the other rodent species almost always began the attacks by biting the prey without grasping it with their paws, and only afterwards did they seize it from the mouth with their forepaws, at which point they started handling it. Only rarely did they use the forepaws to seize the insects before biting. As revealed by the complexity ratio, the hunting ethogram of the rat was more complex than that of any other species. Indeed, the rat displayed a highly versatile manipulation of the prey item and less predictable transitions between stereotype elements compared to all the other eight species, for which the sequence of the behavioral stereotypes was instead more uniform and predictable. Basically, for the rats, the hunting sequence had the greatest inter-trial variability and flexibility. As for the frequency of lethal behavior, 100% of the shrews killed the insect. Among rodents, the frequency of killing behavior was highest for the Tuva silver vole (81.1% of the individuals killed the insect) and the black-striped mouse (80.8%), while it was lowest for the narrow-headed vole (39.1%) and the Djungarian hamster (40%). The killing frequency of the rat was intermediate (51.9%). The hunting complexity reported by this study for the Norway rat is consistent with what had been previously found in a study that compared the cricket-hunting behavior of Norway rats and gray short-tailed opossums (*Monodelphis domestica*). In that study, the rats usually started the attack by biting rather than seizing with the paw (Ivanco et al., 1996). Attacks starting with an attempt to seize with a forepaw were uncommon and generally unsuccessful. Typically, the rats first used their mouth to capture the cricket and then employed their paws to reorient the cricket in such a manner that the cricket's head could be eaten first. In contrast, the opossums usually started a catch with a forepaw and more commonly consumed the abdominal region first. The post-capture behavioral sequence was more complex in rats than in opossums. Prey handling was almost absent in the opossums, which almost never used their paws after a catch. In contrast, the rats extensively employed their digits to manipulate the captured insects. Overall, both the studies by Ivanco et al. (1996) and by Reznikova et al. (2019) observed that, compared to the other tested species, the rat's predatory ethogram featured a greater variety of motor acts and a greater number of correctional movements.

In addition to the four mentioned species of hamsters, predatory behavior upon insects has been investigated in golden hamsters (*Mesocricetus auratus*) as well (Polsky, 1974, 1975, 1976, 1977a,b,c, 1978a,b, 1979; Langley and Knapp, 1984; Langley, 1985, 1986a, 1987). Laboratory tests have shown that golden hamsters kill both locusts (Polsky, 1974, 1975, 1976, 1977a,b,c, 1978a,b, 1979) and crickets (Langley and Knapp, 1984; Langley, 1985, 1986a). Typically, when presented with an insect, golden hamsters orient toward the target, approach it, seize it with the forepaws, take a sitting position and eat the captured insect starting with the head (Langley, 1987).

While both carnivorous and omnivorous rodents may prey upon insects, the type of killing behavior is different. Surplus prey-killing is a killing behavior typical of carnivores, occurring when a predator encounters a group of potential prey animals, kills many and eats just a few (Kruuk, 1972; Curio, 1976; Short et al., 2002; Lucherini et al., 2018; Chopin-Rodríguez et al., 2022). Grasshopper mice, carnivorous rodents, if presented with 25 crickets simultaneously, killed all of them (Langley, 2008). Moreover, when presented with 40 crickets in sequence, the grasshopper mice killed all of them, even though they consumed only the first 6–7 (Langley, 2008). In contrast, omnivorous rodents do not show surplus prey-killing (Langley, 2021).

In addition to crickets, grasshoppers and cockroaches, other insects that are hunted by rodents include ants, weevils and beetles. For example, black-striped mice are skilled ant-killers, killing and eating up to 4 ants per minute (Panteleeva et al., 2013). Wild-caught house mice, evaluated in prey selection tests, attacked and ate live earthworms, moth larvae, weevils and slugs (Smith et al., 2002). Grasshopper mice efficiently kill stink beetles, notwithstanding their defensive spraying (Langley, 1981a, 1994). Deer mice (*Peromyscus maniculatus*) too are capable of killing stink beetles, although less efficiently than grasshopper mice, as they are deterred by the antipredator spray defense (Parmenter and MacMahon, 1988; Langley, 1994).

Slowly-flying insects such as butterflies are also killed by rodents. In nature, monarch butterflies (*Danaus plexippus*) are attacked and consumed by the black-eared mouse (*Peromyscus melanotis*), the Mexican volcano mouse (*Neotomodon alstoni*), the Mexican vole (*Microtus mexicanus*), the Aztec mouse (*Peromyscus aztecus*) and Sumichrast's harvest mouse (*Reithrodontomys sumichrasti*) (Brower et al., 1985; Glendinning et al., 1988; Glendinning, 1990; Glendinning and Brower, 1990; Weinstein and Dearing, 2022). Among these rodents, black-eared mice are so specialized on a monarch-based diet that an average individual of just 20 g can consume up to 40 monarchs per night, without being deterred by the bitter taste of the cardenolides contained in the monarchs, and without being affected by the toxic effects of these potent sodium pump inhibitors, which usually cause emesis and heart failure within minutes (Glendinning et al., 1988; Glendinning and Brower, 1990; Glendinning, 1992).

Behavioral video monitoring of free-living rodents revealed that the peacock butterfly (*Inachis io*) and the small tortoiseshell butterfly (*Aglais urticae*) are killed by the yellow-necked mouse (*Apodemus flavicollis*), the wood mouse (*Apodemus sylvaticus*) and the bank vole (Olofsson et al., 2011). Interestingly, during evolutionary history, butterfly-mouse prey-predator interactions must have been very frequent, as peacock butterflies developed a specific antipredator defense against small rodents, based on the production of sonic and ultrasonic sounds that make them flee (Olofsson et al., 2012). Laboratory-staged encounters confirmed the effectiveness of this acoustic antipredator defense, showing that yellow-necked mice and wood mice were more likely to flee from sound-producing peacock butterflies than from sound-disabled peacock butterflies (Olofsson et al., 2012).

A group of tropical American rodents known as crab-eating rats (genus *Ichthyomys*) has specialized in capturing and eating small invertebrates from slow-flowing watercourses and their banks, such as crustaceans and aquatic insects (Voss et al., 1982; Voss, 1988; Santillán and Segovia, 2013; Brito et al., 2016; de Córdova et al., 2020; Langley, 2021). This group of semiaquatic crab-killing rodents includes the common crab-eating rat (*Ichthyomys hydrobates*), the Ecuadorian crab-eating rat (*Ichthyomys orientalis*), Pine's crab-eating rat (*Ichthyomys pinei*), Pittier's crab-eating rat (*Ichthyomys pittieri*), Stolzmann's crab-eating rat (*Ichthyomys stolzmanni*), and Tweedy's crab-eating rat (*Ichthyomys tweedii*). Among these rodents' main predatory targets, there are freshwater crabs from the *Pseudothelphusidae* family. Notably, crab-eating rats have developed a very specific and strategic killing behavior to overcome these crustaceans, which are far from being harmless. Indeed, Pseudothelphusid crabs are endowed with large pincers (the chelae), which they raise and use as defense when endangered by the approach of a potential predator. Pittier's crab-eating rat, for example, swiftly darts at the crab, repeatedly launching attacks with its forepaws, until it manages to pin to the ground one of the crab's claws with a forepaw, and then bites it off at the base, continuing to do this with all the moving claws, so that it can then safely consume the body (Voss et al., 1982). The masseter muscles of this crab-eating rat provide a strong biting force to the incisors, which this species uses to bite off the limbs at their articulation with the body and to penetrate the crabs' hard exoskeleton, while the molars are employed for masticating the soft tissues (Voss, 1988). The voraciousness of this species toward crabs is remarkable. For instance, a captive individual, in just 2 days, killed and ate 13 *Neopseudothelphusa fossor* about 4 cm wide and one *Eudaniela ranchograndensis* 5.4 cm wide (Voss et al., 1982). Smaller invertebrates are also eaten by crab-eating rodents. For instance, analysis of the stomach content of Stolzmann's crab-eating rat revealed the presence of *Corydalus* (dobsonfly) larvae, *Macrobrachium* shrimp and *Artystone trisibia* isopods (Brito et al., 2016). Analogously, invertebrates have been found in the stomach content of the common crab-eating rat (Voss et al., 1982), Tweedy's crab-eating rat (Voss et al., 1982; Voss, 1988) and Pine's crab-eating rat (de Córdova et al., 2020). The Ecuadorian crab-eating rat was considered a subspecies of Stolzmann's crab-eating rat, under the name *Ichthyomys stolzmanni orientalis* (Voss, 1988), and very likely also includes invertebrates in its diet, similar to Stolzmann's crab-eating rat.

In the Indo-Austral Archipelago (IAA) of South-Eastern Asia, a group of vermivorous shrew rats and shrew mice have specialized in hunting and eating earthworms, which represent their main dietary component (Martinez et al., 2018; Rowe et al., 2016; Langley, 2021). Such IAA vermivorous rodents include 18 species from 9 genera (*Chrotomys, Crunomys, Echiothrix, Hyorhinomys, Melasmothrix, Paucidentomys, Rhynchomys, Soricomys* and *Tateomys*). Remarkably, these vermivores have evolved a distinctive olfactory capability, having olfactory turbinals that are larger and more complex than those of any other carnivorous or omnivorous rodent (Martinez et al., 2018), allowing them to detect underground worms more efficiently. Moreover, they have evolved teeth that enable them to employ specific strategies to kill worms. For instance, the Central Sulawesi echiothrix (*Echiothrix centrosa*) has lower incisors that are abductable (i.e., that can be separated) and can be used to kill worms in three ways (Musser and Durden, 2014). Worm-impaling can be obtained in two manners: by keeping the lower incisors adducted (close together), this vermivore can form a single, wide piercing lance; on the other hand, by keeping the incisors abducted (separated), it can form a fork that pierces the worm in two more distant points, allowing a more secure hold. Alternatively, as a third method, when the echiothrix finds thinner worms, it can collect them between the abducted incisors, using the teeth as a clamp.

Rodents from the Dipodoidea superfamily also kill and eat invertebrates, with some species, such as the long-eared jerboa (*Euchoreutes naso*), being primarily insectivorous. Long-eared jerboas, which inhabit the deserts of Mongolia and northern China, are bipedal rodents with massive ears that are longer than their head (Dulamtseren et al., 2006). They employ their exceptionally large ears for auditory location of insects, and are capable of aerial capture of flying insects by performing powerful leaps into the air, facilitated by their strong, kangaroo-like hindlimbs. Stunning video footage of the long-eared jerboa hunting and performing an aerial catch of a moth in the darkness has recently been shown in the BBC documentary *Asia* narrated by Sir David Attenborough (Green, 2024). Insects represent 95% of the diet of long-eared jerboas (Sokolov et al., 1996; Dulamtseren et al., 2006).

In Africa, the semiaquatic rodents from the *Colomys* and *Malacomys* genera prey upon aquatic invertebrates, mainly insects (Kingdon, 1974; Dieterlen and Statzner, 1981; Kerbis Peterhans and Patterson, 1995; Giarla et al., 2021; Langley, 2021). These two genera form a group of rodent waders that hunt in the water without swimming, but rather by staying perched on their elongated stilt-like hind feet and capturing prey from shallow forest streams. These rodents are endowed with a dense array of vibrissae on the muzzle, which they use to detect the invertebrates that move in the water. This rodent group comprises the African water rats (*Colomys goslingi, Colomys eisentrauti, Colomys lumumbai* and *Colomys wologizi*), the big-eared swamp rat (*Malacomys longipes*), Cansdale's swamp rat (*Malacomys cansdalei*) and Edward's swamp rat (*Malacomys edwardsi*). The predatory behavior of the African water rat *Colomys goslingi* has been studied extensively by Dieterlen and Statzner, who captured an individual from the wild and observed it in the laboratory for a period of over 2 years (Dieterlen and Statzner, 1981). These researchers found that the rat was nocturnal and that it hunted, killed and ate worms, tadpoles and even guppies in shallow water, avoiding instead deeper water.

Within the rodent suborder of Anomaluromorpha, the Anomaluridae (or scaly-tailed flying squirrels) are a family of 6 species belonging to the genera *Anomalurus* and *Idiurus*, all native to central Africa. Scaly-tailed flying squirrels are endowed with a flying membrane (the patagium) connecting the forelimbs with the hindlimbs, which they can extend to glide from one tree to another (Corbin and Cordeiro, 2006; Panyutina et al., 2020). Primarily herbivorous, these gliding rodents supplement their diet by hunting insects (Rosevear, 1969; Julliot et al., 1998; Kingdon, 2015; Panyutina et al., 2020). For example, analysis of the gastric content of Lord Derby's scaly-tailed squirrel (*Anomalurus derbianus*) revealed great quantities of isopterans and hymenopterans: about 40 termites (mostly soldiers) and over 100 ants (from at least three species) in a single individual (Julliot et al., 1998).

Remarkably, predation upon invertebrates is present in rodents as evolutionarily distant as Myomorpha (the so-called mouse-like rodents) and Hystricomorpha (the so-called porcupine-like rodents). Indeed, until very recently, porcupines were regarded as strictly herbivorous (Woods, 1973; Bruno and Ricciardi, 1995; Hafeez et al., 2011). Nevertheless, a study from our decade revealed that crested porcupines (*Hystrix cristata*) actually eat insects regularly in the cold season (Mori et al., 2021). More specifically, analysis of fecal samples of free-living Italian porcupines, collected over a period of 4 years, showed that insects were present in 7% of the samples and the insect-positive samples always belonged to the February-March period of the year. Insects represented 10% of all diet elements. The eaten insects included dipteran larvae, coleopteran larvae of false wireworms belonging to the Tenebrionidae family, and the hymenopterans *Lasius emarginatus* and *Messor capitatus*. These results highlight how rodents can be seasonal predators, which during the cold season enrich their trophic niche with protein-rich food. Analogously, another hystricomorph rodent, the Central American agouti (*Dasyprocta punctata*), while being primarily herbivorous (mainly frugivorous), during the season of fruit scarcity can supplement its diet by killing and consuming crabs, as has been observed in the mora swamp forest of the Osa Peninsula in Costa Rica (Smythe, 1978; Mora et al., 2024).

Importantly, the invertebrate-killing behaviors mentioned up to now are aimed at feeding. Consequently, they can be classified as predatory behaviors. Indeed, the insect-killing instinct of rodents appears to be strongly related to their diet. A comparative study measuring the frequency of lethally attacking a live insect (a cricket) ranked rodent species based on their degree of predatory behavior, finding that in the laboratory test both the frequency of killing and the latency to eat were closely related to the species' degree of carnivory in nature (Timberlake and Washburne, 1989). In particular, the frequency of insect-killing and the rapidity in beginning consumption were greatest in a strongly carnivorous species (the Northern grasshopper mouse *Onychomys leucogaster*^6^) and progressively decreased moving toward herbivory, from omnivorous species (such as the Eastern deer mouse *Peromyscus maniculatus* and the white-footed mouse *Peromyscus leucopus*) to predominantly herbivorous species (like the Mongolian gerbil and the Cairo spiny mouse *Acomys cahirinus*), with the hispid cotton rat *Sigmodon hispidus*, which showed no hunting behavior at all, at the bottom end of the ranking.

Invertebrate-eating among rodents is not infrequent. William Michael Langley, who has been studying rodent predatory behavior for over 45 years, has recently compiled a list of 63 carnivorous rodent species that feed on insects, earthworms, crustaceans and terrestrial non-insect arthropods (Langley, 2021). Among the other rodent species, the vast majority opportunistically incorporate animals (mainly insects) into their diet and can thus be regarded as omnivorous (Landry, 1970), with animal matter representing a secondary food source. Both carnivorous and omnivorous rodents kill and eat invertebrates, being obligate predators or opportunistic predators, respectively. For carnivorous rodents, prey animals are the primary source of food, whereas for the opportunistically predating rodents they are mostly a diet supplement. While relatively few rodents are primarily carnivorous, the vast majority of the existing rodent species are predominantly herbivorous animals that opportunistically predate on invertebrates. Overall, it can hence be estimated that invertebrate-killing is relatively common among rodent species.

The common shrew was classically classified as a type of mouse. Early naturalists included it in the group *Mus* and used the name *Mus araneus* (spider mouse) for it, in reference to the poisonous bite of this animal^7^. However, in the early 19th century, shrews were classified as Insectivora, a mammalian order distinct from Rodentia. Between the late 1990s and the beginning of the 21st century, molecular analyses clarified that Insectivora is not a monophyletic group. Consequently, many species were moved out of the order, while the remaining species, including all soricid shrews (also known as true shrews), were redefined as belonging to the order Eulipotyphla, which is the currently accepted taxonomic classification for true shrews (Stanhope et al., 1998; Douady et al., 2002; Douady and Douzery, 2003).

Shrews are small mammals (including the world's smallest mammal, the Etruscan shrew *Suncus etruscus*) with a high energy expenditure and a consequently high calorie requirement (Genoud, 1988). To meet their elevated metabolic needs, shrews are voracious predators of arthropods, including beetles, ants, spiders and millipedes (Churchfield et al., 2004). Interestingly, shrews' predation is not limited to terrestrial arthropods but targets aquatic arthropods as well. Moreover, shrews can hunt and kill arthropods that are as big as themselves or even bigger. For example, the Mediterranean water shrew (*Neomys milleri*), with a typical body length of 6–8 cm, has recently been observed hunting, killing and eating crustaceans up to 10 cm long, more specifically white-clawed crayfish (*Austropotamobius pallipes*), freshwater crayfish endowed with two large chelae (Ballanti and Nappi, 2025). Impressively, the Mediterranean water shrew can hunt not just from the dry land (by clinging to the shore with the hindlimbs and dipping the head and the forelimbs), but also in the water. Regarding the aquatic predation, this shrew can not only fish out the prey item by walking in shallow water (1 cm), but it can also dive and perform underwater captures. In one of the observed episodes, a Mediterranean water shrew probed the submerged environment by dipping its head twice. Then, where the water was 10 cm deep, it dived, disappearing underwater for a few seconds and subsequently emerging with a crayfish of 4 cm in its jaws, which it then carried away to a sheltered spot. As shrimps fight back with their claws, the water shrew first launches attacks to amputate the two chelae, and then grabs the clawless shrimps by the cephalothorax to drag them away. Furthermore, these shrews have a toxic saliva that paralyzes the bitten prey, allowing them to win fights against opponents bigger than themselves. In the final stages of the observed predation episodes, the bitten crayfish appeared to be partially paralyzed, which made them easily transportable. Interestingly, this semiaquatic shrew species shows a hunting behavior remarkably similar to that of the semiaquatic crab-eating rats of the genus *Ichthyomys*. Indeed, both are capable of capturing from the water crustaceans that they then consume. Moreover, when fighting against clawed crustaceans, the killing technique is strikingly the same: first they amputate the chelae, using their incisors to bite the articulation between the limb and the carapace, and only when the opponent is clawless do they start consuming the body.

### 2.2 Vertebrate-killing by rodents

**Figure 2:**
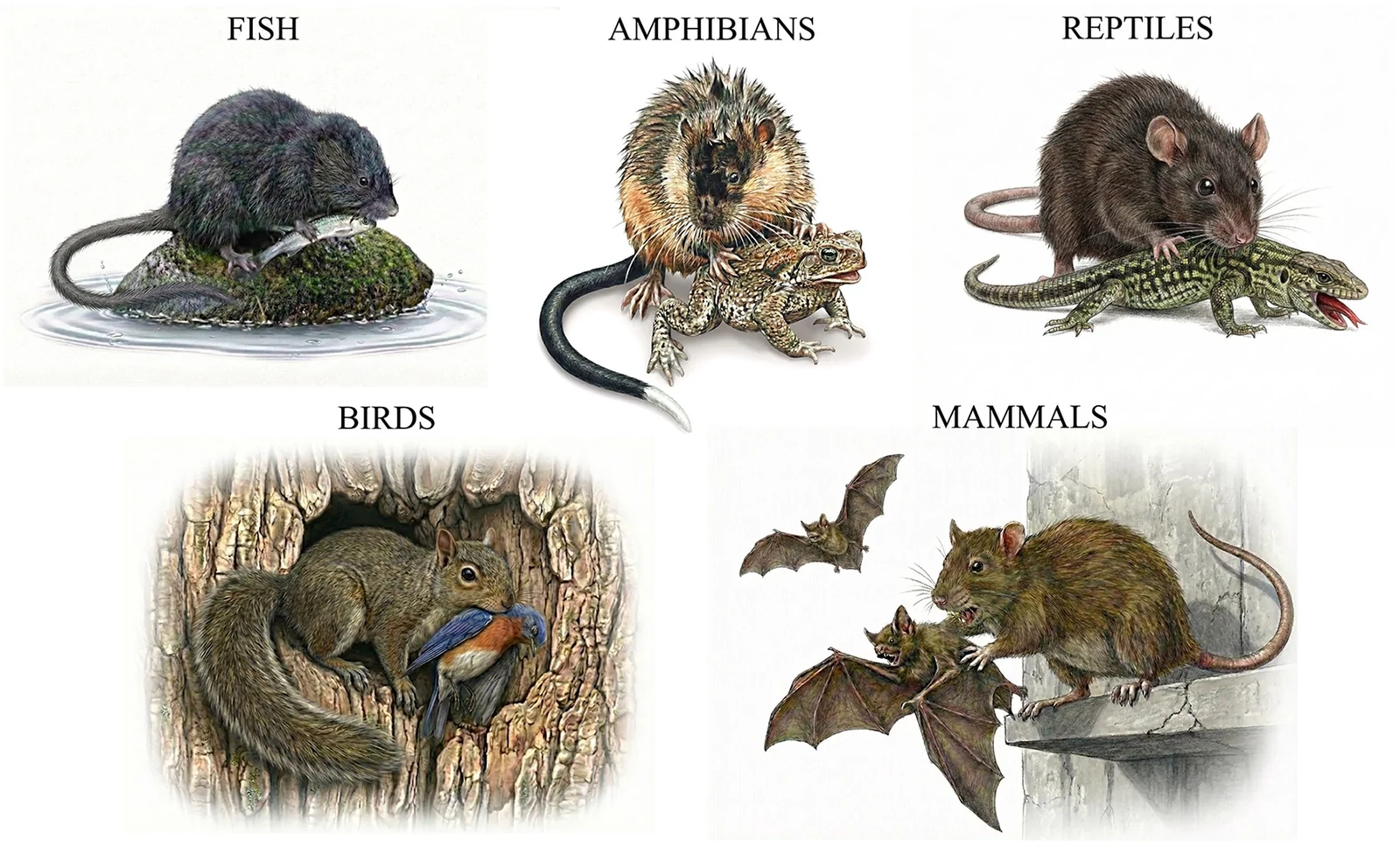
Predatory vertebrate-killing by rodents. Fish: a Las Cajas water mouse (*Neusticomys orcesi*) predating on a fish. Amphibians: a rakali (*Hydromys chrysogaster*) predating on a toad. Reptiles: A black rat (*Rattus rattus*) predating on a lizard. Birds: an eastern gray squirrel (*Sciurus carolinensis*) predating on a bluebird. Mammals: a Norway rat (*Rattus norvegicus*) predating on a bat. Image created by Rafal Stryjek and Raffaele d'Isa.

Remarkably, rodent predatory behaviors are not limited to invertebrates, but may target even vertebrates. All major groups of vertebrates (fish, amphibians, reptiles, birds, and mammals) can be killed by rodents (Figure 2). In this section, we examine in detail predatory vertebrate-killing by rodents, devoting a specific subsection to each prey category.

#### 2.2.1 Fish-killing by rodents

Some rodents have evolved the ability to capture, kill and eat underwater vertebrates, more specifically fish. The most prominent rodent predator of fish is the Australian water rat (also known as rakali) *Hydromys chrysogaster*. A recent analysis of fecal samples collected from wild Australian water rats found that fish DNA was present in 92.9% of the samples (Sanders et al., 2024). The most frequent fish species was the common carp (*Cyprinus carpio*), present in 78.6% of the samples, while the most consumed fish group was that of mosquitofish (*Gambusia affinis* or *Gambusia holbrooki*), present in 85.7% of the samples.

In the American continent, the so-called fish-eating rats (genus *Daptomys*) are a group of neotropical semiaquatic rodents specialized in carnivory. According to current knowledge, this group includes 7 species, namely Ferreira's fish-eating rat (*Daptomys ferreirai*), Musser's fish-eating rat (*Daptomys musseri*), Princess Nunash's fish-eating rat (*Daptomys nunashae*), Oyapock's fish-eating rat (*Daptomys oyapocki*), the Peruvian fish-eating rat (*Daptomys peruviensis*), the Venezuelan fish-eating rat (*Daptomys venezuelae*) and the new *Daptomys species nova*, yet to be named, very recently discovered by an exploratory expedition in Peru (Pacheco et al., 2025). *Daptomys* rats belong to the tribe of Ichthyomyines (in Latin *Ichthyomyini*), a larger group of semiaquatic carnivorous rodents, mostly found in the tropical highlands of Middle America and northern South America (Voss, 2015), which comprises over 20 species subdivided into 7 genera (*Anotomys, Chibchanomys, Daptomys, Ichthyomys, Incanomys, Neusticomys* and *Rheomys*) (de Córdova et al., 2020; Salazar-Bravo et al., 2023; Zeballos et al., 2025; Sánchez-Vendizú et al., 2026). The aforementioned crab-eating rats also belong to this tribe (they are the genus *Ichthyomys*).

The name of the *Ichthyomyini* rodent tribe means “fish-rodents”, in reference to their peculiar diet. However, while all the rodents within this tribe surely predate on aquatic animals, in spite of the tribe's name and in spite of 6 species of this tribe bearing “fish-eating” in their name, it is actually unclear whether these rodents actually predate just on aquatic invertebrates or on fish as well. As the behavior of these rodents is still very understudied, even for the species containing “fish-eating” in their common name there is currently scarce documentation that they are actually capable of capturing fish. Nevertheless, fish-eating is confirmed for at least 8 Ichthyomyine rodents: the Chibchan water mouse (*Chibchanomys trichotis*), Goldman's water mouse (*Rheomys raptor*), Thomas's water mouse (*Rheomys thomasi*), Underwood's water mouse (*Rheomys underwoodi*), the Las Cajas water mouse (*Neusticomys orcesi*^8^), Pittier's crab-eating rat, Stolzmann's crab-eating rat and Tweedy's crab-eating rat (Starrett and Fisler, 1970; Voss et al., 1982; Voss, 1988; Barnett, 1997; Jenkins and Barnett, 1997; Langley, 2021).

Notably, for one Ichthyomyine species there is even clear videographic evidence of fish-killing and fish-eating: the Las Cajas water mouse (Barnett, 1997; Jenkins and Barnett, 1997). Indeed, in 1994 this water mouse was filmed during fish predation by the nature documentarians James Clare and Teresa Clare, and the footage was broadcast in a BBC documentary the following year.^9^ The mouse can be seen entering the water, swimming while searching for prey with its vibrissae, finding a fish, proficiently capturing it in its mouth and finally bringing it out of the water for consumption on dry land. Interestingly, the Clares kept the specimen of the video in captivity for 4 months, before releasing it back into the wild. The Las Cajas water mouse was kept in a seminatural environment that allowed assessment of its fish-killing behavior. This seminatural environment, measuring approximately 1.5 m x 1.5 m, had vegetation from Las Cajas, a burrow and an artificial water system comprising a waterfall and a stream with fish. Over the course of the 4 months, the animal, predominantly nocturnal, regularly killed and ate several fish per night, and its weight grew from 18 g at its arrival to 27 g at its release.

The predatory behavior of Pittier's crab-eating rat has been described by the zoologist Robert Voss and colleagues. In 1979, a specimen was collected from a mountain stream in the evergreen rainforest of Venezuela and brought to the Universidad Central de Venezuela, where its behavior was studied (Voss et al., 1982). Every night, the rat was placed in a glass terrarium filled with local soil and comprising an artificial pool with shallow water (1–2 cm deep). The rat voluntarily entered the pool and systematically lowered its muzzle so that the ends of its vibrissae could touch the bottom of the pool, after which it moved through the water by sweeping the vibrissae as if scanning the substrate. This peculiar searching behavior was never observed outside the water. Upon touch by the vibrissae, live prey items (which had been provided by the experimenters) were readily attacked and killed. Interestingly, the size of the prey influenced the killing strategy. Small prey animals (15–20 mm) were seized by using the forepaws and were then bitten repeatedly until subdued. The prey items were eaten while remaining in the water, while the rat was rearing on its hindlimbs and using the forepaws to hold the prey. Nymphs of dragonfly (Libellulidae), caddisfly (Hydropsychidae), stonefly (Perlidae) and dobsonfly (Corydalidae) were killed and eaten in this manner. On the other hand, large prey animals (50–65 mm) were blocked on the pool's bottom with the forepaws and bitten repeatedly until dead or moribund. Then they were carried out of the water and eaten on dry land. Fish, more specifically goldstripe characins (*Creagrutus beni*), and tadpoles were killed in this way. Being slippery, fish were hard to catch but, repeatedly, the persistence of the rat in its fishing behavior inexorably made it successful. After having brought a moribund/dead fish out of the water, the rat used the forepaws to keep it well pressed to the ground, and then bit the fish with the incisors, performing powerful backward jerks of the head to tear apart the flesh of the fish, so that the prey item could be consumed.

In 2021, USA Today published surprising videographic evidence of Norway rats predating on fish (USA Today, 2021). In Changsha (China), rats were proficiently swimming in a lake toward a group of koi fish that had come close to the water surface to eat some pieces of bread. The rats entered the school of fish and repeatedly attacked them, with one rat catching a fish and then swimming out of the school, carrying away the prey in its mouth.

#### 2.2.2 Amphibian-killing by rodents

Australian rodents from different genera have been found to kill and eat toads. Such toad-killing rodents include the dusky rat *Rattus colletti*, the pale field rat *Rattus tunneyi*, the grassland mosaic-tailed rat *Melomys burtoni*, and the Australian water rat (Cabrera-Guzmán et al., 2015; Parrott et al., 2020). Australian water rats, in particular, preferentially target large cane toads (*Rhinella marina*) over smaller ones and, after turning the toads onto their back and performing a precise thoracic incision, they selectively consume non-toxic organs such as the heart and the liver, while skillfully extracting and avoiding the toxic gall bladder (Parrott et al., 2020).

In North America, grasshopper mice are also capable of killing toads. Experimental testing showed that wild-caught southern grasshopper mice (*Onychomys torridus*) kept in field enclosures, when offered tadpoles or adults of the spadefoot toad (*Scaphiopus hammondi*), attacked and devoured the toads within minutes, usually instantly (Horner et al., 1965). The tadpoles and the adults were attacked by the grasshopper mice with equal rapidity.

Norway rats kill frogs, as extensively demonstrated in the 1970s by laboratory tests in which frogs were presented to the rats by the experimenters (DeSisto and Huston, 1970; Bernstein and Moyer, 1970; Huston and DeSisto, 1971; Bandler, 1970, 1971; Bandler and Chi, 1972; Jimerson and Reis, 1973; Bernard, 1974, 1976; Barr et al., 1976; Gay et al., 1977; Kilbey et al., 1977). Interestingly, it has been found that neither upregulation nor downregulation of testosterone signaling altered this behavior (Bernard, 1974, 1976). This indicates that frog-killing by rats is an androgen-independent killing behavior, corroborating the idea that frog-killing is a form of predatory killing rather than an expression of competition-related social aggressiveness (Bernard, 1974, 1976). Frog-killing was instead suppressed after administration of tripelennamine (an antihistaminic drug blocking H1 receptors) and imipramine (a tricyclic antidepressant upregulating serotoninergic and norepinephrinergic signaling), along with any type of frog-attacking behavior (Barr et al., 1976).

#### 2.2.3 Reptile-killing by rodents

The black rat (*Rattus rattus*) can kill lizards. In Tenerife, the biggest of the Canary Islands (Spain), black rats feed on the Canarian spotted lizard (*Gallotia intermedia*), a large-sized reptile that can reach a total length of 40 cm, to the point that they represent a strong threat to the survival of this already critically endangered endemic lizard, as highlighted by a recent study (López-Darias et al., 2024). More specifically, this study found that 27% of the sampled rats had consumed lizards and that there was a positive correlation between rat density and the decline of the lizard population.

House mice can kill lizards as well (Norbury et al., 2023; Monks et al., 2024). For example, in Mana Island (New Zealand), after the removal of all other introduced mammals, mice had a dramatic impact on the population of McGregor's skink (*Oligosoma macgregori*), which was rescued only after the complete eradication of mice (Newman, 1994; Miskelly, 2023; Monks et al., 2024). In North America, grasshopper mice can kill lizards too (Bailey and Sperry, 1929; Egoscue, 1960; Sherbrooke, 1991). From staged encounters, it was seen that even a 30-day juvenile northern grasshopper mouse could readily kill a brown-shouldered lizard (*Uta stansburiana*) and eat it (Egoscue, 1960). Similarly, southern grasshopper mice, in 30-min staged encounters, reliably showed predatory attacks toward lizards, which several times were fatal (Sherbrooke, 1991). In particular, the attacks of the grasshopper mice were lethal in 43% of the encounters with the roundtail horned lizard (*Phrynosoma modestum*) and in 20% of the encounters with the Texas horned lizard (*Phrynosoma cornutum*).

In the Chagos Archipelago (Indian Ocean), black rats predate on eggs of the green turtle (*Chelonia mydas*), which they access by digging tunnels obliquely in the sand (Stokes et al., 2024). Black rats are capable of finding the eggs notwithstanding the fact that they are buried. Once they locate a nest, after removing the eggs from the clutches and carrying them through the tunnels, the black rats consume them on the sand surface. East of Australia, on Surprise Island (New Caledonia, French overseas collectivity in the Pacific Ocean), after seasonal seabird populations had left the island, researchers observed predation by the black rat on green turtle hatchlings, which likely become a prominent food resource in the season when the seabirds are away (Caut et al., 2008). More recently, researchers presented the first videographic documentation of predation by the black rat upon green turtle hatchlings (Gronwald et al., 2019). On the atoll of Tetiaroa (French Polynesia, French overseas collectivity in the Pacific Ocean), video monitoring of a green turtle nesting site showed that the rats repeatedly visited the site in the 2 days before the emergence of the hatchlings, and started sniffing and digging in the sand 1 h before the emergence. When a hatchling began to emerge, the rat bit its head and pulled it out of the nest, before carrying it away.

#### 2.2.4 Bird-killing by rodents

Birds are not exempt from rodents' attacks either. According to a recent evaluation of threats to seabirds, rats from the *Rattus* genus predate on over 100 species of seabirds, while house mice and deer mice target with predatory attacks 22 seabird species, 20 of which are *Procellariiformes* such as the albatrosses (Dias et al., 2019). Most of these rodent attacks on seabirds concern chicks. For example, on Gough Island (a sub-Antarctic island belonging to the Tristan da Cunha archipelago in the Southern Atlantic Ocean, classified as Important Bird Area), researchers captured video footage of mice attacking chicks of the Tristan Albatross (*Diomedea dabbenena*), the Great Shearwater (*Ardenna gravis*) and the Atlantic Petrel (*Pterodroma incerta*) (Wanless et al., 2007). However, from the same island and from Marion Island (a sub-Antarctic island belonging to the Prince Edward Islands in the Southern Indian Ocean), there is also evidence of house mice killing adults of large-sized seabird species, more specifically the wandering albatross (*Diomedea exulans*) and the Tristan albatross (Connan et al., 2024). Recently, on Marion Island, researchers have recorded direct videographic evidence showing a house mouse attacking an adult wandering albatross (Risi et al., 2025). In the same study, it was found that 13 adult wandering albatrosses had mouse-inflicted wounds (mainly bites on the scalp and on wing joints). Of these attacked albatrosses, 23% died from the mouse bites. Importantly, the attacks of the mice had fatal consequences for the chicks as well. Indeed, the nests with injured adults had a breeding success rate of 45%, much lower than the island-wide average, which was 61% (Risi et al., 2025). At Cape Davis, the area of Marion Island where adult albatross carcasses with mouse bites were first discovered in 2023 (Connan et al., 2024), the breeding success was as low as 28% (Risi et al., 2025).

Sciurids are capable of killing birds and opportunistically do so to eat them (Callahan, 1993). Predation on birds has been reported for several sciurid species. Sciurids' targets comprise both wild birds and human-bred birds from farms. Reports based on direct observations include the eastern gray squirrel (*Sciurus carolinensis*) entering a tree hollow and coming out with a captured bluebird (genus *Sialia*) in its mouth (Bailey, 1923), the 13-lined ground squirrel (*Ictidomys tridecemlineatus*) preying upon a young domestic chicken (*Gallus gallus*) (Bailey, 1923), Franklin's ground squirrel (*Poliocitellus franklinii*) killing and eating ducks (Sowls, 1948), the California ground squirrel (*Otospermophilus beecheyi*) capturing a newborn quail and killing “a fairly large chicken” (Sumner and Dixon, 1953), the rock squirrel (*Otospermophilus variegatus*) capturing and eating young wild turkeys (*Meleagris gallopavo*) (Cook and Henry, 1940), the round-tailed ground squirrel (*Xerospermophilus tereticaudus*) chasing, killing and consuming house sparrows (*Passer domesticus*) (Bradley, 1968), the least chipmunk (*Neotamias minimus*) predating on tree swallows (*Tachycineta bicolor*) (Lederle et al., 1985), the red squirrel (*Sciurus vulgaris*) killing a house sparrow (Nero, 1987), the fox squirrel (*Sciurus niger*) predating on a juvenile blue jay (*Cyanocitta cristata*) (Shaffer and Baker, 1991), and the European ground squirrel (*Spermophilus citellus*) catching, killing and eating a juvenile Eurasian tree sparrow (*Passer montanus*) (Kachamakova et al., 2022).

Squirrels can predate on bird eggs as well. For instance, California ground squirrels have been seen breaking and sucking quail eggs within the quail nest (Sumner and Dixon, 1953), and the fox squirrel has been observed climbing without hesitation to a nest of white-winged doves (*Zenaida asiatica*), picking up the eggs, piercing them and eating the contents (Colson et al., 2011). A 3-year assessment of duck egg predation in Delta Marsh (an extensive marsh area located south of Lake Manitoba in Manitoba, Canada) found that Franklin's ground squirrel destroyed so many duck nests that it was the second most prominent egg predator after crows (Sowls, 1948). In particular, of 206 monitored nests, 47% were destroyed by predators. Crows destroyed 21% of the duck nests, Franklin's ground squirrels 12%, skunks 8% and unknown predators 6%. Nevertheless, some of the nests were on water or on islands, and were therefore inaccessible to the squirrels. Considering specifically the duck nests that were in the squirrels' habitat (the dry-land nests), it was found that Franklin's ground squirrels destroyed 19% of them, a proportion comparable to that of crows. Interestingly, as Franklin's ground squirrels appear to be incapable of breaking duck eggs with their mouth alone, they carry the egg away from the nest and then employ a specific technique to access the egg's content (Sowls, 1948). They search for an appropriate location and, with their nose, they push the egg into a depression or against a mound of earth or clump of grass where the egg cannot roll away. At that point, they place their forelimbs over the egg, pull it underneath their body and block it in that position by bringing forward their hindlimbs. Finally, holding the egg still in this way, they push the egg backwards with their teeth and forelimbs, while simultaneously pressing the egg forward with the hindlimbs, repeating the simultaneous pressures until the egg breaks.

Glirids also hunt birds. In particular, common dormice (*Muscardinus avellanarius*) can enter the arboreal nests of small hole-nesting birds and eat entire clutches of their eggs (Juškaitis, 1995). Other glirids predate not only on the eggs, but also on the birds themselves. In spring, birds can represent up to 26% of the total diet of the edible dormouse (*Glis glis*) (Juškaitis et al., 2015) and, strikingly, up to 86% of the diet of the forest dormouse (*Dryomys nitedula*) (Juškaitis and Baltrunaite, 2013). A recent study based on individual tracking of transponder-tagged edible dormice in northeastern Czechia confirmed that their predation on cavity-nesting birds is not incidental (Adamík et al., 2025). Indeed, 23 visits by edible dormice to bird nests were recorded, and it was found that in 78% of the cases the dormice displayed nest predation. Of the total number of predation events, 78% were on bird nestlings, while 22% were on bird eggs. In the vast majority of the cases (89%), the predator was a male. The edible dormice depredated nests of the collared flycatcher (*Ficedula albicollis*), the great tit (*Parus major*), the coal tit (*Periparus ater*) and the Eurasian nuthatch (*Sitta europaea*). Non-cavity nesting birds may also be targeted by dormice. Recently, researchers have obtained direct videographic evidence of edible dormice predating or attempting predation on eggs and nestlings of two open-nesting songbirds, the common blackbird (*Turdus merula*) and the Eurasian blackcap (*Sylvia atricapilla*), in a forest in eastern Czechia (Adamík and Weidinger, 2024).

Bird-killing is not limited to Myomorpha and Sciuromorpha, but has been observed even in Hystricomorpha, more specifically in agoutis. While predominantly herbivorous, these rodents can opportunistically supplement their diet by killing and consuming vertebrates. For instance, the Central American agouti was observed predating on the clay-colored thrush (*Turdus grayi*) in Costa Rica (Ramírez-Calvo et al., 2017). In particular, a male agouti was initially feeding on fruits, but when it saw a clay-colored thrush, after moving stealthily, it pounced on and captured the thrush with its forelimbs. The rodent used its incisors to kill the bird and then, with the prey item in its mouth, it moved a few meters away. There, it began to consume the bird, which was done in the same posture that the agouti adopts when feeding on seeds: sitting on its hindlimbs and manipulating the food item with its forelimbs. The agouti went on eating for about 15 min, after which it had consumed several parts of the bird, including the muscles, the viscera and even the head. Also in Costa Rica, a free-living Central American agouti was seen chasing a female black-breasted wood-quail (*Odontophorus leucolaemus*) and her four chicks (Mora et al., 2024). Notwithstanding the quails' escape, the agouti managed to capture a chick and ate it. When it finished, the agouti attacked again, grabbing another chick and consuming it as well. Moreover, the Brazilian agouti, also known as red-rumped agouti (*Dasyprocta leporina*), has been seen preying on the brown dove (*Zenaida macroura*) and on eggs of the chicken (*Gallus domesticus*) with the embryonic chick inside (Jones et al., 2019). When wild-caught or captive-born Azara's agoutis (*Dasyprocta azarae*) were exposed, as a test, to eggs and chicks of red junglefowls (*Gallus gallus*), as well as to eggs and adults of Japanese quails (*Coturnix japonica*), both groups showed immediate predation, suggesting an instinctual zoophagic response (Monteiro-Filho et al., 1999). Consistently, free-living Azara's agoutis have been observed, through camera traps, ingesting quail eggs left by the researchers near the border of the forest (Guimaraes-Silva et al., 2021).

#### 2.2.5 Mammal-killing by rodents

The most well-studied case of vertebrate interspecific killing by rodents is that of rats killing mice. This mouse-killing behavior, also known as muricide, has been extensively investigated in the laboratory. The trailblazer of this line of inquiry, which began in the 1950s, was the French neurobiologist Pierre Karli (1926–2016), with his work in the Psychobiological Laboratory of Curt Paul Richter (1894–1988) at the Johns Hopkins Hospital in Baltimore. Curiously, Karli's creation of this sub-field of rodent behavioral testing originated from a serendipitous discovery, as recalled by Karli himself later on (Karli, 1979). In 1954, after winning a fellowship from the Rockefeller Foundation, Karli came to the USA to perform behavioral research in Richter's laboratory. Upon his arrival, Karli was provided with a great number of rats and mice but a limited number of cages. He therefore housed mice and rats together, and soon discovered that some rats killed the mice while others did not. When he relayed this observation to Richter, the latter replied by encouraging Karli to investigate further: “Look, Pierre, you may well have here the topic to work on for the twelve months to come. If this mouse-killing response proves to be a stable all-or-nothing response, it may well provide a useful behavior for the study of the biology of aggression” (Karli, 1979). Karli then started to design specific experiments to investigate the mouse-killing behavior of rats, which led him to the publication of a work that became one of the biggest classics of this field (Karli, 1956).

In this work, Karli showed that the mouse-killing response could be easily evoked in a testing session, just by placing a mouse in the home-cage of the rats. Both wild and laboratory rats exhibited the mouse-killing behavior. However, the frequency of this behavior was much higher in wild rats (70%) than in laboratory rats (12%). This is likely an effect of domestication. As laboratory rats have been created from pet rats that had undergone artificial selection to make them more suitable as companion animals (d'Isa, 2025b), it is possible to imagine that domesticated rats have undergone selection not just for physical characteristics (such as whiteness, which conveyed the idea of cleanness), but also for behavioral traits such as tameness and reduced mordacity. Moreover, Karli found that the likelihood of mouse-killing was comparable between sexes (male wild rats: 68%; female wild rats: 70%; male laboratory rats: 13%; female laboratory rats: 11%).

Karli also noted a constancy in the response to the mouse: rats that killed in the first 3 trials never stopped, while rats that did not kill in the first week never killed later on. The main method of mouse-killing was by biting the cervical area and breaking the spinal cord. Occasionally, other killing methods were employed. In order of frequency, the killing methods were lethal biting of: spinal cord (89%; cervical: 65%; thoracic 13%; lumbar 11%), ventral side (7%) and head (4%). All rats consumed the body of the killed mouse. Nevertheless, Karli observed a striking difference between wild and domesticated rats in the body parts they preferred to consume. Wild rats ate, in order of frequency, thoracic viscera (81% of the trials), abdominal viscera (71%) and the brain (50%). In contrast, laboratory rats showed a marked preference for immediately opening the skull and eating the brain. In 95% of the trials, laboratory rats ate nothing but the brain.

In the same work (Karli, 1956), Karli investigated the neural basis of the mouse-killing response. In killer rats, amygdalectomy abolished mouse-killing behavior. Amygdala-ablated killer rats never killed a mouse again and “lived peacefully with the mice, never displaying the least sign of hostility toward them”. Conversely, rats that had never killed the test mouse, after bilateral lesion of the frontal cortex, started killing. Furthermore, Karli explored the effect of gestation on mouse-killing. A group of non-killer female laboratory rats were placed, after mating, in a cage with a mouse. In the first weeks post-mating, the rats showed no sign of interest in the mouse, and the two species lived on opposite sides of the cages. However, in the peripartum period, the behavior of the females changed abruptly. Notably, 1–2 days before delivery, 70% of the females began to display very active maternal behaviors, such as retrieval of the mouse, holding it between the forelimbs and grooming it. The remaining 30% started to show the same maternal behaviors toward the mouse after parturition. Finally, Karli tested the effect of food deprivation on mouse-killing. Non-killer rats (both laboratory and wild) could not be induced to kill the mouse by food deprivation, even if this lasted 3 days. A group of laboratory non-killer rats was offered mouse meat, which they ate, and were then presented with a live mouse in their home-cage. Strikingly, the vast majority of the rats (75%) preferred to starve to death rather than kill the mouse, for which they instead displayed a “friendly interest”, as Karli defined it.

After Karli's groundbreaking works (Karli, 1956, 1958, 1959, 1961), a great number of studies were conducted on rats' mouse-killing behavior, especially between the 1960s and 1980s (Heimstra and Newton, 1961; Whalen and Fehr, 1964; Heimstra, 1965; Myer, 1967, 1971; Baenninger, 1970; Baenninger and Baenninger, 1970; Smith et al., 1970; Panksepp, 1971; Avis and Treadway, 1971; Di Chiara et al., 1971; Miley and Baenninger, 1972; Paul, 1972, 1975; Lonowski et al., 1973, 1975; Grant et al., 1973; Paul and Posner, 1973; O'Boyle, 1974; Barr et al., 1975; Paul and Kupferschmidt, 1975; Rossi, 1975; Mack and Mandel, 1976; Gay et al., 1977; Logan et al., 1977; Marks et al., 1977; Adamec and Himes, 1978; Albert and Brayley, 1979; Bandler and Vergnes, 1979; Giammanco and La Guardia, 1979a,b; Albert et al., 1981, 1982a,b, 1983, 1984a,b, 1985a,b, 1986; Albert and Walsh, 1982; Bammer and Eichelman, 1983; Kemble et al., 1985; Onodera and Ogura, 1985; Molina et al., 1986, 1987; Tani et al., 1987; Takaoka et al., 1988; Isel and Mandel, 1989; Yamamoto et al., 1989; Giammanco et al., 1990a,b; Onodera et al., 1991, 1993; Bac et al., 1995, 1998; Tadano et al., 1997; Ueda et al., 1999; Ho et al., 2004; Chiang et al., 2008). The researchers after Karli explored the effects of previous experiences, anatomical lesions and pharmacological agents on rat mouse-killing.

For instance, the fact that wild rats kill mice more often than laboratory rats led to the hypothesis that this difference was due to a greater competition experienced by the wild rats in their natural habitat before capture (Heimstra and Newton, 1961). A competition training protocol was therefore developed, in which two food-deprived rats were placed in a box containing a cylindrical food source which could be accessed by only one rat at a time (Heimstra and Newton, 1961). Experiments employing this training protocol confirmed that previous experience with inter-rat food competition increases the likelihood of mouse-killing (Heimstra and Newton, 1961; Whalen and Fehr, 1964; Heimstra, 1965). In particular, rats that were food-deprived and underwent inter-rat food competition killed 4 times more mice than rats that underwent only food deprivation (Heimstra, 1965). Previous diet can also affect mouse-killing behavior. Rats maintained on a non-mouse flesh diet (raw beef) did not exhibit increased mouse-killing (Paul, 1975). On the other hand, rats kept on a dead-mouse diet killed mice more frequently than rats maintained on a standard chow diet, and this effect was observed under both food deprivation and *ad libitum* feeding conditions (Paul, 1975). Food-deprived rats showed a four-component attacking ethogram, comprising chasing, carrying, pawing and biting mice (Adamec and Himes, 1978). However, with experience, the attacking ethogram simplified to a two-component attack, pawing-biting, which resulted in a rapid and lethal strike. Hunger itself appeared to modulate the attacking ethogram. Indeed, food satiation reduced mouse-killing behavior and reinstated attacks comprising three components: chasing, pawing and biting. Nevertheless, re-depriving the rats of food led them to switch again to the rapidly lethal pawing-biting attacks.

Lesional studies provided evidence regarding the neurobiological substrates of rat mouse-killing. For example, lesions of the medial hypothalamus, the lateral septum or the region ventral to the anterior septum all induced strong home-cage mouse-killing, with 85–100% of the rats in each group showing this behavior in the test at 2 days post-surgery, and 50–70% in the test at 21 days (Albert and Brayley, 1979). Strikingly, lesions of the lateral septum or the region ventral to the anterior septum also led the rats to kill and eat juvenile (150–200 g) male rats, a behavior which was never observed in the unlesioned rats, suggesting an abnormal increase of the predatory instinct in the lesioned rats (Albert et al., 1981).

Finally, the effects of several pharmacological manipulations on mouse-killing were investigated. Ethanol did not induce non-killer rats to kill mice (Bammer and Eichelman, 1983). Only at high doses, causing motor impairments, did ethanol reduce muricide in killer rats (Bammer and Eichelman, 1983). It hence appears that ethanol does not actually have a specific effect in reducing the muricidal instinct and that the observed reduction in the killings was just related to drug-induced ataxia. On the other hand, rat mouse-killing has been found to be truly inhibited by several neuropharmacological agents, including the serotonin precursor L-5-hydroxytryptophan (5-HTP) (Di Chiara et al., 1971), the monoamine oxidase inhibitor pargyline (Di Chiara et al., 1971), GABA (Mack and Mandel, 1976), taurine (Mack and Mandel, 1976), fluoxetine (Molina et al., 1987), citalopram (Molina et al., 1987), S-adenosyl-L-methionine (Yamamoto et al., 1989), the monoamine oxidase inhibitors moclobemide, cimoxatone and toloxatone (Isel et al., 1988), and the monoamine oxidase inhibitors minaprine, CM 30366 and SR 95191 (Isel and Mandel, 1989).

In addition to Norway rats, black rats carry out muricide as well (Lidicker, 1976; Bridgman, 2012). Typically, the removal of black rats from a territory where house mice live or roam leads to bursts in the number of house mice. For instance, in New Zealand, suppression operations on invasive black rats in the forest were followed by increases in house mouse abundance (Brown et al., 1996; Bridgman et al., 2013). On Santiago Island (Galápagos Islands, Ecuador), the removal of black rats led to the competitive release of house mice (Harris and Macdonald, 2007). In Buck Island (a Caribbean island belonging to the Virgin Islands of the United States), the successful eradication of introduced black rats was followed by a dramatic rise in the population of house mice (Witmer et al., 2007). In experiments with wild-caught black rats exposed to wild-caught house mice in dyadic encounters with a wire-mesh screen dividing the animals, it was seen that the black rats behaved aggressively toward the mice, and that their attacks were not preceded by threat behaviors (such as raised hackles, lateral display and hip throwing), unlike the attacks exhibited during dyadic intraspecific encounters (Bridgman et al., 2013). Moreover, when euthanized mice were offered to the black rats, the black rats attacked the mice and ate them at least partially (Bridgman et al., 2013). For rats with *ad libitum* access to food, the average time to start consuming the mouse was just 22.5 s. Hunger further lowered this latency. For food-deprived rats, the average latency was, impressively, 1.4 s. The immediate, abrupt and warningless nature of the attacks, as well as the fact that the attacks were related to feeding, altogether indicate that the attacking behavior of black rats upon house mice had a predatory nature.

Recently, infrared video monitoring of the Segeberger Kalkberg cave in northern Germany documented the first systematic predation by free-living Norway rats on bats in an urban hibernaculum (Gloza-Rausch et al., 2025). Remarkably, the rats were not only capable of ground-hunting, through which they killed landed bats, but were also capable of aerial interception, capturing bats while they were in flight. In these aerial interceptions, the rats stood upright on their hindlimbs, maintaining balance through their tail, and employed their forelimbs to skillfully capture, manipulate and restrain bats that flew next to them. Impressively, these captures were made in complete darkness, so the rats were likely detecting the bat through tactile cues from their vibrissae, including the vibrations generated by the air currents produced by the bat's flight. Such skilled and versatile hunting strategies highlight the behavioral plasticity of rats in urban ecosystems.

Rats are not the only rodent predating on mammals. Franklin's ground squirrel has long been known for its flesh-eating tendencies, for which we have several historical reports (Bailey, 1893, 1926; Johnson, 1922; Seton, 1929; Howell, 1938; Sowls, 1948; Landry, 1970). This squirrel's predation is not limited to bird eggs and birds (see section 2.2.4), but extends to mammals as well. The zoologist Vernon Orlando Bailey (1864–1942), examining the stomach of a wild-caught individual, found several eastern deer mice inside it (Bailey, 1893). The botanist Arthur Monrad Johnson (1878–1943) provided a detailed account of the case, witnessed while he was crossing the forest of western Aitkin County (Minnesota, USA), of an adult Franklin's ground squirrel killing a young rabbit, as large as the squirrel, and devouring it (Johnson, 1922). The zoologist Arthur Holmes Howell (1872–1940), in his beautifully illustrated and now classic monograph on North American ground squirrels, relayed: “Individuals of this species have been known to kill and eat wild mice and a young rabbit, to rob a meadowlark's nest, to kill a wood pewee, to capture small chickens, and to eat hens' eggs” (Howell, 1938). The zoologist Lyle Kenneth Sowls (1916–2002) analyzed the stomach contents of 178 Franklin's ground squirrels, finding that 20% contained animal flesh, and, more specifically, 10% contained mice, 3% hares, 3% toads, and 4% unspecified carrion (Sowls, 1948). Sowls then performed a test on wild-caught Franklin's ground squirrels housed in a pen (Sowls, 1948). If live mice were introduced into the pen, they were always killed with swiftness and eaten. On the other hand, cold dead mice were always left uneaten. The live mice were invariably attacked by being seized from the head and then bitten. By holding the mouse in their forepaws, the squirrels peeled back with care the mouse's skin, which they do not consume. Analogously to Sowls' pen-housed squirrels, when a wild-caught Franklin's ground squirrel, that had been captive for a month, received in its home-cage a mouse as a companion, it attacked, killed and ate the mouse within 5 min (Seton, 1929).

Predatory killing of heterospecific mammals by sciurids has subsequently been reported for a certain number of species, but mostly through single serendipitous observations. Examples include a white-tailed antelope squirrel (*Ammospermophilus leucurus*) killing and consuming a pocket mouse (Morgart, 1985), California ground squirrels killing and eating several northern broad-footed moles (*Scapanus latimanus*) (Trulio et al., 1986), strong indirect evidence of Arctic ground squirrels (*Urocitellus parryii*) killing and consuming 6 radio-tagged northern collared lemmings (*Dicrostonyx groenlandicus*) (Boonstra et al., 1990), and Alpine marmots (*Marmota marmota*) in the Nuremberg Zoo (Germany) occasionally predating on newborns of Alpine ibex (*Capra ibex*) (Böhm and Landmann, 2021).

However, widespread and video-documented predation of a mammalian species by a squirrel species has been reported only very recently. In June and July 2024, a study based on visual monitoring of marked California ground squirrels in Briones Regional Park (California, USA) observed 74 instances of squirrels killing and/or consuming California voles (*Microtus californicus*) (Smith et al., 2025). For the 51 cases for which individual identification was possible, it was seen that 27 featured different individuals. The highest percentage of vole hunters was among females. In particular, vole-hunting occurred in 43% of adult females, 23% of juvenile females, 15% of adult males and 15% of juvenile males. As this vole-hunting behavior by California ground squirrels was associated with a decadal peak in vole abundance, it has been interpreted by the authors of the study as an example of behavioral flexibility in foraging.

Predation on mammals can be observed in Hystricomorpha as well. In captivity, when a painted spiny pocket mouse (*Heteromys pictus*) accidentally escaped from its cage and entered the cage of a Central American agouti, it was promptly killed and eaten by the agouti (Smythe, 1978). More than once, when captive Central American agoutis were offered beef meat, they ate it, preferring cooked meat to raw meat (Smythe, 1978), just like another closely related agouti species (Roth-Kolar, 1957). In Costa Rica, a free-living Central American agouti was seen carrying in its mouth a nine-banded armadillo (*Dasypus novemcinctus*), one of the largest species of armadillos (Mora et al., 2024). After the researchers followed the animal to take pictures, the agouti dropped the armadillo, which could be inspected. The armadillo was dead and had deep wounds inflicted by the agouti's incisors. The armadillo was a young individual, which could have made the killing easier for the agouti.

## 3 Antipredatory killing

Prey animals can counter predation with a set of antipredatory behaviors. Rodents' most common antipredatory behaviors are escape and avoidance. However, some rodents respond to predation with defensive aggression. Antipredatory aggression by rodents is rarely lethal and is generally simply a tactic to gain space and time for escape. Nevertheless, some rodent species are capable of antipredatory killing, i.e., the killing of potential predators in order to avoid predation.

Porcupines are the prototypical rodent group showing antipredatory killing. When exposed to a potential predator, crested porcupines (*Hystrix cristata*) show an agonistic ethogram which includes four major components: (a) quill erection; (b) tail-rattling; (c) stomping of the hind feet; (d) attack (Mori et al., 2014; d'Isa et al., 2026). The porcupine's attacking behavior is quite peculiar. Basically, the animal turns its back toward the opponent and runs backwards into it, piercing the target with the erected quills (backward attack). A similar attack may be performed by the porcupine also by running laterally (sideways attack). Notably, porcupines can kill their opponent not just actively, but also passively, when their opponents attack and impale themselves against the quills. Nevertheless, both the active and the passive killings rely on an active behavior of the porcupine: quill erection. Moreover, porcupines' turning backwards further facilitates the self-impalement of their opponents. Therefore, in the case of porcupines, even passive killing actually has an active component.

In Africa, porcupines are predated by big cats such as lions (*Panthera leo*) and leopards (*Panthera pardus*). However, porcupines can also kill their feline predators. The zoologist Julian Kerbis Peterhans and colleagues have provided a detailed review of lion-porcupine interactions, including 10 cases in which lions were killed by porcupines, either crested porcupines or Cape crested porcupines (*Hystrix africaeaustralis*) (Kerbis Peterhans et al., 2019). Leopards can equally be fatally injured by porcupines. For instance, in 2012, the wildlife photographer Marga Stevens documented, through a series of photographs in Kruger National Park (South Africa's largest nature reserve), the case of an adult leopard killed by a Cape crested porcupine, with quills embedded in the facial area, the neck, the chest and the scapular region (Lombard, 2016; Bush 24, 2017).

In the American continent, the North American porcupine (*Erethizon dorsatum*) is predated by several aerial predators. Nevertheless, predation attempts can prove fatal for these raptors. In fact, North American porcupines can kill several species of avian predators, including the golden eagle (*Aquila chrysaetos*), the bald eagle (*Haliaeetus leucocephalus*), the rough-legged hawk (*Buteo lagopus*), the common raven (*Corvus corax*) and Steller's jay (*Cyanocitta stelleri*) (Katzner et al., 2015). Lethal wounding of these species typically involved quills perforating the throat, the heart and the intestine. Moreover, great horned owls (*Bubo virginianus*) have been found moribund after interactions with North American porcupines, including one that was discovered with 112 quills in its body (Wiley, 1969). Interestingly, quill-withdrawal experiments revealed that North American porcupines are endowed with an antipredatory mechanism that facilitates the release of the quills when the quill's root is pushed back into the porcupine's skin, as happens during a predator's attack (Roze, 2002). For this reason, the porcupine's quills have been defined as “smart weapons” (Roze, 2002, 2006, 2012). Notably, this facilitation mechanism occurs only in the quills that have been erected by the porcupine, which underscores the importance of the behavioral response of the porcupine for the successful killing of an attacker (Roze, 2002). Indeed, experimental tests measuring the tension required to remove quills from the porcupine's skin, after that they had been struck with a piece of styrofoam, found that the force required for quill extraction was significantly lower in aroused porcupines (with erected quills) than in anesthetized porcupines (with relaxed quills).

The aversion of porcupines to dogs has been known since at least the Renaissance. In Europe, this aversion has been observed in the crested porcupine. For example, after the Italian mathematician and physician Gerolamo Cardano (1501–1576) saw a captive porcupine in Pavia^10^, he relayed that, while the animal was easily managed by the person conducting it, it showed the greatest hostility to dogs (Cardano, 1550). The Swiss naturalist Conrad Gessner (1516–1565) wrote that porcupines use their quills to wound dogs (Gessner, 1551). Consistently, the Italian physician and botanist Pietro Andrea Mattioli (1501–1578) reported^11^ that porcupines often injured the dogs of the hunters (Mattioli and Dioscorides, 1554, 1555). At present, in Italy, it has been reported that free-living porcupines reacted with quill erection in 100% of the interspecies encounters with hunting dogs, which escalated into attacks in 50% of the cases (Mori et al., 2014). In 83% of these fights, the dogs ended up injured or killed. The same Italian study also reported carcasses of dead predators, such as the red fox (*Vulpes vulpes*) and the Eurasian badger (*Meles meles*), killed by porcupine quills.

North American porcupines are also capable of killing mammals. Like crested porcupines, North American porcupines have long been known for their dangerousness to dogs. In the 1720s, the French naturalist and physicist René Antoine Ferchault de Réaumur (1683–1757) wrote an essay on the North American porcupine, based on the observations described in the letters that he had received from Michel Sarrazin (1659–1734), who at that time was physician of the King in Quebec (de Réaumur, 1729). Sarrazin relayed how in Canada these porcupines were injuring and even killing hunting dogs. Interestingly, Sarrazin highlighted how the structure of the quills made them easily releasable from their root, unlike the spines of hedgehogs. Remarkably, by performing microscopical examinations of porcupine quills, Sarrazin discovered that, at their sharp end, porcupine quills are endowed with backward-facing indents which make them more difficult to extract once they penetrate the flesh of an opponent, a feature which makes the quills more lethal. In our time, the Western College of Veterinary Medicine of the Veterinary Teaching Hospital (WCVM-VTH) of the University of Saskatchewan in Saskatoon (Saskatchewan, Canada), examining the veterinary records of a 5-year period (1998–2002), found 296 cases of dogs with quill injury (Johnson et al., 2006).

Indian porcupines, also known as Indian crested porcupines (*Hystrix indica*), are the largest rodents in the Indian subcontinent (Chakravarthy et al., 2013). Analogously to the common and the North American porcupines, Indian porcupines can also cause fatal wounds to other mammals. At the end of the 18th century, the British soldier, composer and writer Thomas George Williamson (1758/9–1817), who spent 20 years in India as part of the British army (from 1778 to 1798), observed that Indian porcupines were injuring hunting dogs with their quills, which were penetrating deeply into the dogs' flesh and detaching from the porcupines' skin (Williamson, 1807).

British hunter and author Edward James Corbett (1875–1955), who killed man-eating tigers and leopards in India for over three decades, explained in his best-selling memoir *Man-eaters of Kumaon* that the quill-caused injuries after fights with Indian porcupines, and the consequent quill-induced disability, were one of the main reasons for tigers and leopards becoming man-eaters (Corbett, 1944). Typically, these man-attacking felines ended up being killed by humans. Consequently, the deaths of these big felines can be seen as being indirectly caused by the injuries inflicted by the porcupines. Subsequently, the Anglo-Indian feline hunter Kenneth Anderson (1910–1974) confirmed Corbett's claim that big felines can turn into maneaters after having been injured by porcupines. In particular, in *The Spotted Devil of Gummalapur*, Anderson recounted the case of a leopard that had become a man-eater after sustaining serious injuries from porcupine quills (Anderson, 1954). This leopard slaughtered 42 persons in the area of the village of Gummalapur, before being killed by Anderson himself. Post-mortem examination showed that the leopard had porcupine quills embedded in a paw, which was likely impairing its ability to hunt non-human prey.

Indian porcupines can even directly cause the death of their attackers. For example, in 2012, in the Anamalai Tiger Reserve, a tiger was found dead with two porcupine quills penetrating its heart (Jeganathan, 2012). In 2015, in the Periyar Tiger Reserve, a dead tiger of about 15 years was discovered with porcupine quills embedded all over its body (The Hindu, 2015). One quill had pierced the tiger's tongue and lower jaw. The abdomen and the limbs were also pierced by quills. In 2022, in the Katarniaghat Wildlife Sanctuary, a tiger was found dead and the autopsy revealed that it had died from internal bleeding caused by porcupine quills that had perforated its thoracic cavity (Siddiqui, 2022).

The American herpetologist Henry Sheldon Fitch (1909–2009) reported that California ground squirrels can kill Pacific gopher snakes (*Pituophis catenifer catenifer*), especially young ones (Fitch, 1949). Gopher snakes feed upon California ground squirrels. Analysis of the stomach content of gopher snakes revealed that, of all the different ingested food items, ground squirrels represented the highest percentage of the total food weight (44%). Remarkably, gopher snakes do not find the California ground squirrels only in the open, but often enter their underground burrows. When these underground encounters occur, a fight follows and the snake is often bitten fatally. This outcome in favor of the squirrel is perhaps facilitated by the restricted space of the tunnels that make it more difficult for the snakes to launch their wrapping attack, which they use to capture their target before fatally constricting it. After killing the snakes, the squirrels carry them to the surface and drop the carcasses near the opening of their burrow. On many occasions, Fitch found badly lacerated dead young gopher snakes dropped on ground squirrel burrow mounds, to the point that he estimated that most of the deaths of young gopher snakes were caused by the ground squirrels. Notably, none of the snakes that were found killed on the squirrel burrow mounds had been eaten, indicating that these killings by the squirrels are not related to predation, but rather to antipredatory behavior. To further explore the capability of California ground squirrels to kill snakes, Fitch experimentally staged an encounter by placing a young gopher snake in the cage of a captive female California ground squirrel (Fitch, 1949). The squirrel showed hair-fluffing and started to move cautiously around the snake with slow, tense steps. After this, it struck, by biting the snake on the tail, and then retreated. Shortly after, the squirrel launched another attack, biting the posterior part of the snake. Then, the squirrel grabbed the snake with its forepaws and bit its spinal cord, breaking it, after which it dropped the snake. This action was repeated multiple times, and every time the bite was placed at progressively more rostral positions of the spinal cord, causing an increasing amount of paralysis in the snake. Finally, when the snake was completely paralyzed and defenseless, the squirrel bit through the head and neck of the snake, killing it. Remarkably, the snakes found killed on the squirrel burrow mounds all had injuries resembling the pattern of bites exhibited by the snake neutralized by the captive squirrel, strongly suggesting that those snakes had been killed in the same way. Of these dead gopher snakes, almost all were young, but on one occasion a dead adult, of approximately 60 cm, was also found.

## 4 Social competition-related killing

### 4.1 Competition-related intraspecific killing

Many rodents are highly territorial, meaning that, in nature, they choose a specific home range and consistently defend it against potential conspecific competitors through agonistic behaviors, in particular threats^12^, non-lethal physical attacks and killings. Territorialism is particularly well-known in house mice and Norway rats, which are the most widely employed animal models in biological research (Makowska and Weary, 2021; Domínguez-Oliva et al., 2023; d'Isa and Gerlai, 2023). In the laboratory, rodent territorialism can be studied through colony or home-cage models.

In the colony model, mice or rats are housed within large enclosures in mixed-sex groups. An example is the Visible Burrow System developed by the Blanchards (Robert Blanchard and Dixie Caroline Blanchard), which is provided with tunnels and chambers mimicking the burrow systems created by rodents in nature (Blanchard et al., 1985; Blanchard and Blanchard, 1989). When both sexes are present in the colony, intermale conflict is particularly intense and the mortality of subordinate animals can be strikingly high. Indeed, it has been found that in mixed-sex colonies of Long-Evans rats, within 4 months, 58% of subordinate males died (Blanchard et al., 1985). Interestingly, as many of the dead rats did not show injuries compatible with fatal wounding, it has been hypothesized that dominant rats can kill subordinate rats both directly (through a fatal attack) and indirectly (through social stress causing life-shortening physiological alterations) (Blanchard et al., 1985, 1998, 1995; Herman and Tamashiro, 2017). When 6 male wild-caught rats were introduced, at the same time, in an equally unfamiliar space of 1 m^3^ together with females, within less than 3 months the dominant male killed all the other males (Barnett, 1955). Analogously, in mixed-sex mouse colonies, all subordinate males died within a short period (Kemble et al., 1993).

In the home-cage model, best known as the resident-intruder test, an unfamiliar conspecific (the intruder) is placed in the home-cage of an individually housed animal (the resident). Typically, the resident rapidly attacks the intruder. If the intruder is left in the resident's home-cage for more than extremely brief testing sessions^13^, there is a high risk of mortality for the intruder. In tests with Long-Evans rats, within 4 h, male adult residents killed 42% of male adult intruders and 25% of male adolescent intruders (Thor and Flannelly, 1976). Of all lethally attacked intruders, 25% were killed in the first 10 min. Notably, winning a fight is rewarding to rats and mice, and repeated experiences of fight victories increase the violence of the attacks of the residents, which switch from basic non-lethal aggression, featuring mainly bites to the back and the flanks (where the skin is thicker), to violent attacks on highly vulnerable targets like the throat, the head and the abdomen, which have a higher likelihood of lethality (de Boer et al., 2009; Koolhaas et al., 2013).

The colony and resident-intruder models can also be combined, if an unfamiliar conspecific is introduced into a colony (Blanchard et al., 1975, 1977). In this colony residents-intruder test, the intruder is attacked fiercely. Notably, after the introduction of adult conspecifics, adult Sprague-Dawley colony rats killed 66% of the intruders within 36 h (Blanchard et al., 1975).

Interestingly, among wild house mice, there are intraspecies genetic differences in the propensity to kill conspecifics. In nature, chromosomically diverse populations of house mice exist (Hauffe et al., 2000). For example, in Italy, there are house mice with 11, 12, 13, 14 or 20 pairs of chromosomes (Corti et al., 1986; Miczek et al., 2001; Giménez et al., 2017). The wild variants with 12 and 13 pairs are sympatric, but the population with the 13-pair karyotype has the capability to displace that with the 12-pair karyotype from specific locations (Hauffe et al., 2000; Miczek et al., 2001), as happened in the small Alpine village of Migiondo (Italy) in just a few years (Capanna and Corti, 1982; Hauffe and Searle, 1992). This is likely due to differences in the aggressive behavior of the two karyotypic variants. Indeed, from experimental dyadic encounters between heterokaryotypic individuals (13 pairs vs. 12 pairs), it has been found that resident 13-pair mice had a greater likelihood of attacking the other individual, as well as a considerably lower latency to attack (Capanna et al., 1984; Corti et al., 1989). Fights between resident 13-pair mice and intruder 12-pair mice were initiated by the residents in 86% of the cases, while fights between resident 12-pair mice and intruder 13-pair mice were initiated by the residents in just 57% of the cases (Capanna et al., 1984). Moreover, compared to 12-pair mice, the 13-pair mice were more likely to kill an intruder placed in their home-cage (Capanna et al., 1984). In particular, 12-pair residents killed 5% of the heterokaryotypic intruders and 5% of the homokaryotypic intruders, while 13-pair residents killed 15% of the heterokaryotypic intruders and 25% of the homokaryotypic intruders.

Apart from house mice and Norway rats, other rodents showing paradigmatic killing behavior toward conspecific competitors are grasshopper mice. Indeed, grasshopper mice are extremely territorial. Once established in a territory, these mice exhibit a very peculiar acoustic advertisement behavior to signal their presence. Specifically, grasshopper mice take a bipedal stance, raise their nose to the sky and, with the ears laid back and the mouth wide open, produce a loud and prolonged high-pitched whistle (Hafner and Hafner, 1979; Pasch et al., 2017), which has been traditionally described as howling, with the only difference that it has no rise or fall in its pitch (Bailey and Sperry, 1929). The particular posture taken during vocalization maximizes the reach of this acoustic signal. Moreover, the laryngeal anatomy of grasshopper mice has adapted to facilitate long-distance communication (Pasch et al., 2017). These long-distance vocalizations were originally compared to wolf howls because of the prolonged high pitch of the signal and the head posture taken by the emitter, but it is now believed that they also share the same function as wolf howling. Wolf howls have a double role. On the one hand, they play a crucial role in territory maintenance and distancing of potential competitors (Harrington and Mech, 1979, 1983). On the other hand, they enable mate attraction during the breeding season (McIntyre et al., 2017). Similarly to wolf howls (McIntyre et al., 2017), grasshopper mouse howls reach their maximal frequency of occurrence in the breeding season. In particular, grasshopper mouse howls peak in early spring and continue throughout the summer (Bailey and Sperry, 1929). Notably, grasshopper mouse howls are sexually dimorphic and hence bear information regarding the sex of the emitter (Hafner and Hafner, 1979). Both males and females howl, but males howl more often than females, with a study finding that 77% of the howling individuals were males (Hafner and Hafner, 1979). In addition to spontaneous howls, grasshopper mice emit this vocalization upon detecting the proximity of a conspecific, generally climbing to the highest place that they can reach (such as a log or a rock) before howling (Hafner and Hafner, 1979). Interestingly, it has been seen that howling males had enlarged scrota compared to non-howling males (Horner and Taylor, 1968; Hafner and Hafner, 1979). The breeding-related seasonality, the higher prevalence in reproductively active males and the acoustic sexual dimorphism of the howls have led researchers to hypothesize that grasshopper mouse howls have the function of warning off male competitors and attract females.

Remarkably, Alston's singing mouse (*Scotinomys teguina*), a neotropical insectivorous cricetid belonging to the sub-family of the Neotominae as grasshopper mice, exhibits an identical vocal behavior. In an upright position, with the snout raised and the mouth wide open, it emits loud and long vocalizations, from which it takes its name (Hooper and Carleton, 1976; Burkhard et al., 2023). This long-distance advertisement plays roles in both male-male competition and mate attraction, functioning simultaneously as an aggressive display and a courtship display (Hooper and Carleton, 1976; Fernández-Vargas et al., 2011). Indeed, males sing after exposure to both males and females. Males paired with a female started singing within 5 min (Hooper and Carleton, 1976). After a 3-min exposure to a male, they also sang, although less abundantly than after exposure to a female (Fernández-Vargas et al., 2011). However, the singing behaviors after male-male and male-female encounters were associated with opposite social behaviors during the encounter (Fernández-Vargas et al., 2011). While male-female encounters were characterized by high levels of allogrooming and an almost complete absence of agonism, in male-male encounters the reverse occurred, with 64% of the trials featuring agonistic behaviors such as threats or fights. It hence appears that the singing has opposite roles after an encounter with a male or with a female, being an aggressive signal in the first case and a courtship signal in the second. While further experimental testing is needed, it is highly likely that the howling of the grasshopper mouse, analogously, has the double function of competitor-intimidation and female-attraction.

Grasshopper mice are particularly intolerant of any form of potential social competition and show extremely aggressive territorial behaviors even against members of their own species, which commonly result in the killing of the encountered conspecific. By investigating the outcome of 132 same-sex staged encounters between northern grasshopper mice in a seminatural enclosure, it was found that the subordinate of the dyad was inevitably killed within 3 days of the initial contact, with 70% of the subordinates being killed within the first 24 h (Ruffer, 1968). Intraspecific same-sex killing was observed not only in the males, but also in the females. In either male-male or female-female encounters, a dominance hierarchy was established and the subordinate was killed. Only opposite-sex pairs could live together. Interestingly, the intraspecific encounters always started with circling, but the diameter of the circle was over 36 cm in the same-sex dyads, while it was just 10–15 cm in opposite-sex dyads, revealing an immediate and accurate sex recognition ability. In the seminatural enclosure, any grasshopper mouse in addition to a single male-female pair was systematically killed, without having the chance to survive more than two nights.

In breeding colonies of grasshopper mice, fights are rare and killings even rarer. However, when they occur females are generally the aggressors. In the breeding colony of the University of Utah in Dugway (Utah, USA), where about 1,000 northern grasshopper mice were reared in the period 1953–1957, observations from 43 breeding females showed that in 5 male-female pairs the males were killed by the female (Egoscue, 1960). In each of these cases, the pair mated successfully and lived in harmony until just before parturition, when the female, possibly due to a state of increased anxiety, killed the male. However, this peculiar prenatal killing behavior was rare and the majority of the females continued to live in harmony with the males even during late pregnancy.

Kangaroo rats are castorimorph rodents native to North America, comprising about 20 species belonging to the genus *Dipodomys* (Daly et al., 1984). Kangaroo rats are solitary and territorial rodents that react aggressively to the presence of conspecifics, to the point that they cannot even be maintained in pairs, which poses a serious challenge for captive breeding. Laboratory experiments with chisel-toothed kangaroo rats (*Dipodomys microps*) showed that, if a male was put in the home-cage of a female, or if a female was put in the home-cage of a male, severe aggression occurred in both cases, which led the researchers to cease all attempts to conduct mating trials in the home-cage of one of the two rats (Daly et al., 1984). Mating was possible only in a neutral arena, where 1-h encounters were staged. However, even there, non-receptive females behave aggressively toward the males, and even the receptive females do so after copulation, to the point that the males can be killed if the pairs are not promptly separated by the experimenters. In another species, Merriam's kangaroo rat (*Dipodomys merriami*), 1-h encounters in a neutral arena were not successful in eliciting sexual interest (Daly et al., 1984), so another approach had to be employed. Merriam's kangaroo rats of opposite sex were housed in home-cages that were connected by a tunnel featuring a guillotine door (Daly et al., 1984). When the female reached estrus, the door was opened and the two individuals were granted access to each other. Although this procedure was successful in strongly reducing fighting behavior, even with this strategy, in 5 of 800 linked-cage pairings one of the mates killed the other one, and in 80% of the cases it was the female that killed the male.

In black rats, intraspecific killing of adults (adulticide) was identified a very long time ago. As early as the 1750s, the French naturalist Georges-Louis Buffon (1707–1788) described having witnessed that, in conditions of overpopulation, free-living black rats often tended to kill each other: “Despite cats, poison, traps and baits, these animals multiply so rapidly that they often cause great damage; this is especially true in old country houses, where wheat is stored in the attics, and where the proximity of barns and haylofts facilitates their hiding and multiplication to the point that they reach such great numbers that one would be obliged to unfurnish and move out, if they did not destroy themselves; but we have seen from experience that they kill each other, that they eat each other if hunger presses them; so that, when there is a shortage due to an excessive number, the strongest attack the weakest, open their head and eat their brain first, and then the rest of the carcass”^14^ (French edition: Buffon, 1758; English edition: Buffon, 1791).

Nevertheless, these observations by Buffon were largely neglected for a long time, almost falling into oblivion. Interestingly, modern studies have confirmed the competition-related intraspecific killing behavior of black rats. Indeed, black rats tend to form closed social groups, chasing off or killing unrelated newcomers that invade their territory (Iannucci et al., 2018). For instance, a study monitored, through radio-tracking, a group of alien male black rats introduced from southern Corsica (France) onto the nearby Island of Piana (Italy), as well as a group of resident male black rats (Granjon and Cheylan, 1989). Impressively, within few hours of release, the alien black rats were recognized as foreign and killed by the resident population. Of the 4 introduced alien males, one lost its signal in the first hour of release, while the other 3 moved away from the inland to the shoreline, where their signal was lost within 6 h, and where they were found dead or moribund by the following morning. Signs of bites were found on their bodies. One had bites on its head, while another, which had the body partially devoured, was found in a lentisk bush where it had been seen going to, and from which sounds of violent fighting and cries had been heard. In contrast, the residents were monitored for 6 days without signal losses. The resident rats moved freely across both the inland and the coastline of the island.

Dormice (Gliridae) are a family of rodents that comprises about 30 species across Europe, Africa and Asia. Dormice are endowed with the ability to go into torpor or hibernation when the environmental conditions are excessively unfavorable (for example, due to particularly low temperatures) and, in the most northern parts of their habitat, their hibernation may last up to two thirds of the year (Juškaitis, 2023). In laboratory settings, it has been seen that dormice waking earlier cannibalized their still-hibernating neighbors (Airapetyants, 1983). Such intraspecies killing has been observed to be more frequent in forest dormice (*Dryomys nitedula*) and in garden dormice (*Eliomys quercinus*), less frequent in common dormice (*Muscardinus avellanarius*), and apparently absent in edible dormice (*Glis glis*) (Airapetyants, 1983). This “first to wake, first to kill” behavior can be interpreted as either a form of intraspecific predation or intraspecific competition. Nevertheless, these cannibalistic events have traditionally been interpreted as a way to quickly recover energy after the hibernation-induced weight loss, and hence as a predatory behavior (Airapetyants, 1983; Lang et al., 2025). Anyway, in these cases of cannibalism it is not possible to exclude the possibility that the eaten dormice had died of natural causes. Recently, researchers have published the first visually documented case of intraspecies adulticide in dormice (Lang et al., 2025). In particular, by using camera-equipped nestboxes deployed in the natural habitat of the dormice, a free-living adult garden dormouse was filmed entering a nestbox where an adult conspecific was hibernating, and killing this conspecific. The hibernating dormouse was awakened by the attack of the intruder and tried to fight back but, likely also because of low reactivity due to the abrupt interruption of hibernation, it lost the physical confrontation and was killed. Remarkably, the killed dormouse was not consumed by the killer, suggesting that this killing was not predation-related but competition-related. Based on the rapidity of the attack, and on the fact that the killer dormouse displayed repeated post-mortem attacks on its victim, notwithstanding its evident unresponsiveness, the authors concluded that this intraspecific killing behavior was not an accident consequent to a particularly intense fight, but rather a goal-directed behavior specifically aimed at the destruction of the opponent.

While porcupines are mainly known for their interspecific killing behavior, intraspecific fights may also lead them to inflict serious injuries. In some cases, these competition-related injuries can be fatal. For instance, at the Ljubljana Zoo, after an intermale fight between an older and a younger Indian porcupine, the older male ended up with quills perforating its thoracic wall, which led to its death (Švara et al., 2015).

Naked mole-rats (*Heterocephalus glaber*) are eusocial rodents living in highly organized subterranean colonies (typically comprising 70–80 individuals, but occasionally reaching up to 300) that are based on labor division and led by a queen, similar to the colonies of social insects such as bees (Brett, 1991; Schulze-Makuch, 2019; Toor et al., 2022; Hart et al., 2026). Together with the Damaraland mole-rats (*Fukomys damarensis*), naked mole-rats are the only known eusocial mammals (Jarvis and Bennett, 1993; Roellig et al., 2011; Jacobs and Oosthuizen, 2023). The naked mole-rat queen has a reproductive monopoly (meaning that it is the sole breeding female of the colony) and mates with 1–3 males, the consorts (Jarvis, 1981; Brett, 1991; Jarvis et al., 1994; Khallaf et al., 2026). The members of the non-breeding caste are workers (which fulfill tasks such as foraging and nest-maintenance) or soldiers (which are larger and defend, through fighting, the colony from possible attacks by intruding animals) (Jarvis, 1981; Lacey and Sherman, 1991; O'Riain and Jarvis, 1997; Holmes and Goldman, 2021). As naked mole-rats are xenophobic (intolerant of extracolony individuals) and particularly responsive to the scent of foreign colonies, when there is an intrusion by a foreign conspecific, the colony's soldiers often react by attacking with great violence, which can lead to the death of the intruder (Lacey and Sherman, 1991; O'Riain and Jarvis, 1997; Lewis and Buffenstein, 2016; Buffenstein, 2021; Smith and Buffenstein, 2021; Toor et al., 2022).

Interestingly, naked mole-rats live longer than any other rodent, with a maximal lifespan exceeding 40 years, and many individuals surpassing 30 years (Buffenstein and Amoroso, 2024; Ruby et al., 2024; Park et al., 2025). When compared to similarly sized rodents such as house mice, which generally do not live more than 3–4 years, such a lifespan is especially surprising. Indeed, according to a well-known biological law, across mammalian species there is an inverse correlation between body size and lifespan, with the maximum lifespan potential increasing by 16% for each doubling of the average species body mass (Hulbert et al., 2007). Nevertheless, naked mole-rats live about 5 times longer than their size-corrected life expectation (Ruby et al., 2023, 2024). These extended lifespans provide the colony with a long-lasting social stability. However, when the queen dies (or is removed), the process of queen succession is particularly violent. Indeed, after the loss of the queen, the top-ranking females start a fight to the death to become the new queen (Margulis et al., 1995; Clarke and Faulkes, 1997). Basically, females attempt to emerge as the new queen through physical elimination of the rivals, in an extreme form of intraspecific competition. In certain cases, high-ranking females may attempt a coup and attack an already reigning queen (Smith and Buffenstein, 2021). For example, Clarke and Faulkes relayed the case of a colony where the queen was killed by her own daughter, which was the colony's next largest female after the queen (Clarke and Faulkes, 1997). Matricide is a very rare form of killing among rodents, and currently it has been observed only in naked mole-rats. In another reported case, the death of the queen's only breeding male was followed by a takeover event during which 7 competing females were killed, including the queen (van der Westhuizen et al., 2013). In this case too, the queen was killed by her daughter, which became the new queen. During this takeover event, 5 males were killed as well. In total, the death of the queen's consort led to the killing of more than 35% of the remaining colony members (12 of 33 individuals). Together with grasshopper mice and kangaroo rats, naked mole-rats provide the most extreme examples of intraspecific competition-related killing in rodents. However, compared with the cases of grasshopper mice and kangaroo rats, which kill unfamiliar conspecifics that invade their territory, the case of naked mole-rats is even more extreme, as they kill known members of their own colony.

### 4.2 Competition-related interspecific killing

Sympatric rodent species (i.e., rodent species living in the same geographical area) draw on common resources. When these resources are not overabundant, interspecific competition may arise and, when resources are limited, competition may become particularly high. While some rodents respond to interspecific competition with elusive behaviors, others respond aggressively. Examples of elusive behaviors are deceptive dodging (d'Isa et al., 2024a) or temporal niche switching (Stryjek et al., 2026), which are performed by the black-striped mouse in order to avoid the yellow-necked mouse, a bigger and stronger competitor. Conversely, other rodents respond to interspecific competition with aggression.

According to a classical ethological distinction, predatory attacks have a food-securing function, while aggressive attacks have a competition-countering function (Lorenz, 1966; Eibl-Eibesfeldt, 1970). In more recent classifications, predation has been recognized as a form of aggression, namely predatory aggression (e.g., Miczek, 1983; Nikulina, 1991; Haug and Johnson, 1991; Sandnabba, 1995; Gammie et al., 2003; Siegel et al., 2009; Tulogdi et al., 2015; Haller, 2018; Anderson et al., 2022; Gonzalez-Olvera et al., 2025; Eren et al., 2026). Aggression can hence be subdivided into predatory aggression and competitive aggression. However, while the semantics may be different, the original conceptual difference proposed by the earlier ethologists still remains valid. Indeed, several features distinguish the two behaviors. In particular, the frequency of killing is markedly higher in predation than in competition. Predatory aggression is commonly lethal, while competitive aggression is rarely so. Moreover, while predatory attacks are aimed at bringing the target near for a time longer than the instant of the attack, competition-induced attacks are aimed at distancing. Additionally, predatory aggression is commonly active and the target is often actively searched for; on the contrary, competition-induced aggression is typically reactive and produced in response to chance encounters with potential competitors. Finally, the killed targets are usually eaten in the first type of attack, while this generally does not occur in the second type. When competition-related aggression is lethal, the outcome of the attack is the same as in predation. Nevertheless, even in the case of a lethal outcome, predation and competition-related killings display distinct contextual behavior profiles. In Table 1 we present a list of 10 criteria that can be useful to distinguish predatory and competition-related killings.

**Table 1:** Ten main criteria to distinguish predatory and competition-related killings.

Predation	Competition-related aggression
Typically aimed at feeding	Killed animals may or may not be eaten
Predominantly interspecific	Can be directed with equal likelihood to both conspecific and heterospecific unfamiliar individuals
Aimed at keeping the target close after the attack	Aimed at distancing the target after the attack
Stealthy approach	Unconcealed approach
Abrupt initiation, unpreceded by threats	Often preceded by threats
The target's submission increases its chances of being killed	The target's submission can stop the attack
The target is often actively searched for (active encounter)	The target typically enters the subject's territory by chance (passive encounter)
No signaling or luring signaling	Intimidatory signaling
Null or low autonomic activation	High autonomic activation
Anti-aggressive drugs (such as fluprazine) do not reduce it	Anti-aggressive drugs (such as fluprazine) inhibit it

While competitive aggression is generally less lethal than predation, some rodents kill other rodents in response to interspecific competition. For instance, northern and southern grasshopper mice will kill heterospecific rodents that invade their territory. This has been tested under controlled conditions by introducing heterospecific rodents into the living space of grasshopper mice (Bailey and Sperry, 1929; Egoscue, 1960; Horner et al., 1965; Ruffer, 1968; Pellis and Pellis, 1992). In particular, in a seminal study with wild-caught animals (Bailey and Sperry, 1929), it was found that, upon introduction into its home-cage, the northern grasshopper mouse killed several rodent species, including Gambel's deer mouse (*Peromyscus gambelii*^15^), the Great Basin pocket mouse (*Perognathus parvus*) and the montane vole (*Microtus montanus*). When a live Great Basin pocket mouse was left overnight in a grasshopper mouse's cage together with a dead Gambel's deer mouse (as meat supply), in the morning the pocket mouse was still found killed (Bailey and Sperry, 1929).

Further experiments, with wild-caught southern grasshopper mice individually housed in seminatural enclosures, confirmed the killing instinct of grasshopper mice toward heterospecific rodents (Horner et al., 1965). Indeed, if a little pocket mouse (*Perognathus longimembris*) was released into the enclosure, the grasshopper mouse rapidly attacked, killed and consumed it. The killing technique was rapid and efficient. For example, upon the delivery of the pocket mouse, a grasshopper mouse sprang on it at once, bit the dorsal cervical area at the base of the skull, and immobilized the pocket mouse by pressing its head down on the ground with the forepaws. Holding the target in this way, the grasshopper mouse started chewing the lateral side of the pocket mouse's head and within 3 min it had excavated a 0.6 cm hole in the area of the auditory bulla. Within 10 min, the entire head had been consumed and the pocket mouse's body had been skillfully skinned, by turning the skin back. Then the grasshopper mouse left the inside-out skin uneaten and carried the skinned carcass underground to its burrow. Another rodent species, the western harvest mouse (*Reithrodontomys megalotis*), when released in the enclosure, was killed with similar efficiency, notwithstanding the harvest mouse's agility, and devoured. House mice were killed as well.

Subsequent experiments with wild-caught northern grasshopper mice placed in seminatural enclosures showed that, in staged encounters, the grasshopper mice systematically killed, within 3 h of the first contact, heterospecific rodents of a great variety of species, including the western harvest mouse, the eastern deer mouse, the house mouse, the hispid pocket mouse (*Chaetodipus hispidus*), the white-footed mouse (*Peromyscus leucopus*), Ord's kangaroo rat (*Dipodomys ordii*) and even the hispid cotton rat (*Sigmodon hispidus*), whose individuals could weigh three times as much as the grasshopper mice (Ruffer, 1968). Notably, the grasshopper mice were never killed. Both male and female grasshopper mice killed the other rodent species. However, if a male-female pair of grasshopper mice was exposed to an heterospecific rodent, the male went off to kill the stranger, while the female remained in or next to the burrow.

Egoscue analogously reported that, in staged encounters within their home-cage, northern grasshopper mice killed and consumed all species of smaller sympatric cricetids (such as deer mice) and heteromyids (spiny pocket mice) that were offered to them (Egoscue, 1960). Egoscue also described the grasshopper mice's peculiar way of killing. The most typical killing method was through bites that pierced the skull and sank the teeth into the brain. However, occasionally, the grasshopper mice employed an alternative strategy, and strangled their target to death by seizing it and keeping the forepaws tightly pressed on the target's throat.

Interestingly, the grasshopper mouse's motivation to kill heterospecific rodents appears to be highly instinctual, as well as its ability to kill, as it has been shown that no previous interspecific interaction is required for the first kill. Indeed, laboratory-born northern grasshopper mice that were completely naïve to live prey exhibited strong and efficient killing instincts toward heterospecific rodents from the very first encounter (Pellis and Pellis, 1992). Notably, these subjects were second-generation animals (F2) derived from the laboratory-born offspring (F1) of wild-caught individuals (F0), so they had had no direct experience of prey-killing and no contact with animals experienced in prey-killing. These F2 individuals were individually housed in small (50 x 26 x 30 cm) terraria. When house mice were placed in the subjects' living environment, the grasshopper mice attacked and killed the house mice with exceptional rapidity, the average killing latency being just 73 s.

It is worth highlighting an important remark regarding the grasshopper mouse's killing of competitor rodent species. In many cases, the distinction between predatory killing and competition-related killing is very clear. However, in some cases this distinction becomes more subtle and blurred. In particular, after the killing of competitor heterospecific rodent species, grasshopper mice often eat them (Bailey and Sperry, 1929; Egoscue, 1960; Horner et al., 1965). As we have seen, eating the target is typically a feature of predation. Hence, a question arises: is this killing behavior a form of predation or competition? While this killing behavior may serve both functions, several pieces of evidence support the idea that the grasshopper mouse's killing of heterospecific rodents is primarily a form of competition-related killing, and only secondarily a form of predation. First, grasshopper mice largely rely on insects rather than on mammals for their diet. Scat analysis performed on samples from free-living grasshopper mice found that 100% of the individuals had eaten insects, while just 3% had consumed small mammals (Horner et al., 1965). It therefore appears that grasshopper mice do not need rodents for feeding. Second, grasshopper mice live in areas where there is a very high interspecific competition with omnivorous rodent species. They occur in habitats where the populations of omnivorous rodents are 10 times as numerous as grasshopper mice (Langley, 2008). While these omnivorous rodents may eat either insects or vegetal matter, grasshopper mice rely almost entirely on insects for their diet. Consequently, they have a strong need to protect their food sources from other rodent species. Third, since grasshopper mice live in areas with such a great abundance of heterospecific rodents, if they were interested in predating on them, their presence in their diet should be much higher. Fourth, predators either actively search for prey or they lurk concealing their presence in order to launch an ambush with surprise advantage (Kappeler, 2021). In the case of grasshopper mice, it appears that they do not seek other rodent species, nor do they conceal their presence. On the contrary, they employ auditory and olfactory intimidation signals to distance potential competitors. When predators direct signals toward prey targets, these are typically luring signals. For instance, deep-sea anglerfish (Ceratioidei) possess a bioluminescent bait (the esca) at the end of a specialized dorsal fin (the illicium, or fishing rod), through which they lure small fish and invertebrates that they then eat (Miya et al., 2010; Freed et al., 2019). In contrast, grasshopper mice deter heterospecific rodents from foraging in their habitat by emitting the aforementioned aggressive howling. Moreover, they employ olfactory advertisement of their presence, by producing skunk-smelling feces and by active olfactory marking of the territory, which they obtain by rubbing their body on the ground to deposit a secretion produced by their sebaceous glands (Langley, 2021). Remarkably, it has been observed that both the acoustic and the olfactory advertisement signals are effective in intimidating deer mice, major competitors of grasshopper mice. Compared to white noise, playbacks of grasshopper mouse howls reduced deer mice's efficiency in foraging upon crickets and mealworms (Langley, 2008). Analogously, the presence of the skunk-smelling feces of grasshopper mice, compared to their absence, reduced both the time spent foraging and the number of captured prey items (Langley, 2008). It therefore seems that grasshopper mice kill other rodent species only when the intimidatory signals have failed and, by chance, the heterospecific rodents enter the grasshopper mouse's proximity. Finally, the anti-aggressive drug fluprazine, administered to laboratory mice, inhibited intermale attacks and infanticide, but not predation (insect-killing) (Parmigiani and Palanza, 1991). When administered to grasshopper mice, fluprazine inhibited the killing of house mice, but not cricket-killing (Schultz and Kemble, 1986), suggesting that the mouse-killing behavior of grasshopper mice is a form of competition-induced interspecific aggression rather than predation. Altogether, these facts suggest that the selective pressure for the grasshopper mouse's evolution of a killing instinct toward heterospecific rodents is the reduction of interspecific competition, as previously proposed by Langley (Langley, 2021). Indeed, the combination of highly aggressive (potentially lethal) attacks and intimidatory signaling (presence advertisement) is highly functional in reducing the foraging efficiency of heterospecific rodent competitors.

Golden hamsters also kill house mice (Van Hemel, 1977). Laboratory tests featuring staged dyadic encounters between hamsters and mice showed that 100% of the hamsters attacked the stimulus mouse and 25% of the hamsters killed it. Notably, the carcasses of the mice were almost never consumed by the hamsters. Of 15 killed mice, only 2 were chewed by the hamsters, and even those only partially.

A 6-year study, based on direct observations of free-living marked individuals, found that white-tailed prairie dogs (*Cynomys leucurus*) killed, but did not consume, Wyoming ground squirrels (*Urocitellus elegans*) (Hoogland and Brown, 2016). These killings of heterospecific competitors were performed by 47 different individuals, 19 of which were serial killers. Remarkably, 30% of the female prairie dogs were killers. These female killers had markedly higher annual and lifetime fitness than non-killer females, likely because of a lower interspecific competition for plant food sources.

Yellow-necked mice are particularly aggressive toward sympatric rodents, including congeners (such as the wood mouse and the black-striped mouse), and are highly dominant over them (Hoffmeyer, 1973; Montgomery, 1978; d'Isa et al., 2024a; Stryjek et al., 2026; Kurek et al., 2026). Laboratory studies based on staged encounters within a large indoor pen showed that yellow-necked mice attacked wood mice three times as frequently as the reverse and proved dominant over them in 103 cases, while wood mice prevailed in only 11 cases (Hoffmeyer, 1973). Furthermore, in staged encounters within a testing arena, yellow-necked mice dominated wood mice in 63 out of 70 cases, while wood mice won just one fight (Montgomery, 1978). Our team, in a study monitoring the social behavior of free-living yellow-necked mice and black-striped mice, found that 86.7% of the cases of interspecific encounter featured agonism. In these interspecific agonistic encounters, the yellow-necked mouse was the initiator of the agonism in 100% of the cases, attacking with a latency of less than 2 s in 92.3% of the cases, and being dominant over the black-striped mouse in 84.6% (Stryjek et al., 2026).

Moreover, indirect evidence suggests that yellow-necked mice enter the nests of common dormice and kill them (Juškaitis, 2008, 2014; Zaytseva-Anciferova and Nowakowski, 2012). Common dormice are arboreal rodents that spend most of their active season on trees and leave the arboreal habitat only when they look for a safe place where they can hibernate (Juškaitis and Büchner, 2013; Gubert et al., 2023). Yellow-necked mice are skilled tree-climbers, which can move in the canopy at a height of over 20 m from the ground (Borowski, 1962; Montgomery, 1980; Marsh, 1999). Yellow-necked mice do not employ just underground burrows, but also tree nests (Truszkowski, 1974; Juškaitis, 1997; Marsh, 1999). For example, in Lithuania, if in autumn researchers deploy arboreal nestboxes, yellow-necked mice usually occupy 10–13% of these nestboxes (Juškaitis, 2000). Hence, competition over arboreal nests may arise between yellow-necked mice and dormice. In the autumn of 2007, in Lithuania, 6 dormice were found killed inside the nestboxes and several clues suggested that yellow-necked mice were responsible for these killings (Juškaitis, 2014). First, in that autumn the percentage of nestbox occupation by yellow-necked mice was exceptionally high, reaching about three times the average: 35%. Second, in two of the nestboxes containing killed dormice there were caches of hazelnuts, which are hoarded by yellow-necked mice but not by dormice. Third, for a nestbox where 3 dormice were found killed, after the removal of the dead bodies, it was seen that the new occupant was a yellow-necked mouse. Additionally, a different study found, through laboratory behavioral testing, that yellow-necked mice are attracted by the odor of dormouse nests (Zaytseva-Anciferova and Nowakowski, 2012).

Notably, our team has recently reported the first direct observation of a free-living yellow-necked mouse killing a sympatric free-living rodent, more specifically a bank vole (Kurek et al., 2026). In particular, by monitoring social interactions in infrared camera-equipped chambers placed in the natural habitat of the rodents, we observed yellow-necked mice attacking bank voles until they were dead or apparently dead. Importantly, in both the lethal and the near-lethal attacks, the yellow-necked mice completely ignored the vole after the attack. They did not eat or work the vole's body on site, nor did they carry the body away. Such post-attack indifference toward the vole's body indicates that the killing was not related to predation, but rather to interspecific competition. Neutralization of the competitor was the only apparent aim of the yellow-necked mice's attacks. Remarkably, the yellow-necked mice attacked the voles with an impressively low latency (in all cases within 2 s of the encounter) and defeated them with great ease (within 25 s). Interestingly, during our team's field observations of the behavior of free-living *Apodemus* mice from 2020 to 2025, while we recorded many attacks by yellow-necked mice on black-striped mice, we never saw any of these attacks turn lethal (d'Isa et al., 2024a; Stryjek et al., 2026). This could be due to the remarkable escape skills of black-striped mice compared to bank voles. Indeed, while bank voles can in certain cases be more helpless and scarcely reactive to the attacks (Kurek et al., 2026), black-striped mice are very fast and skilled in their escapes, and they are also capable of employing complex deceptive strategies to dodge hostile opponents (d'Isa et al., 2024a).

## 5 Infanticide

### 5.1 Non-parental infanticide

Non-parental infanticide is the killing of a pup by a conspecific that did not generate it. Non-parental infanticide is widespread across rodents, having been identified in 43 species belonging to 28 different genera (see Table 2 for a complete listing of the species). As can be seen from Table 2, this killing behavior is present in rodents as evolutionarily distant as Hystricomorpha and Myomorpha. In nature, non-parental infanticide by rodents can be so common that it may represent the major cause of juvenile mortality (Sherman, 1981; Hoogland, 1985; Blumstein, 1997). This type of killing behavior is exhibited by both males and females. Male non-parental infanticide has been recorded in 31 rodent species and female non-parental infanticide in 30 species (see Table 2). However, in several species, females are more infanticidal than males, as has been observed in the white-footed mouse, the Eastern deer mouse, the prairie vole (*Microtus ochrogaster*), the Mongolian gerbil, the Columbian ground squirrel (*Urocitellus columbianus*), the black-tailed prairie dog (*Cynomys ludovicianus*) and the northern collared lemming (Ebensperger and Blumstein, 2007).

**Table 2:** Rodent species displaying non-parental infanticide.

Family	Species scientific name	Species common name	Males	Females
Caviidae	*Galea musteloides*	Yellow-toothed cavy		X
	*Hydrochoerus hydrochaeris*	Capybara		X
Dasyproctidae	*Dasyprocta azarae*	Azara's agouti	?	?
Heterocephalidae	*Heterocephalus glaber*	Naked mole-rat	?	?
Muridae	*Acomys cahirinus*	Cairo spiny mouse	X	X
	*Apodemus sylvaticus*	Wood mouse	X	X
	*Clethrionomys glareolus*	Bank vole	X	X
	*Cricetus cricetus*	European hamster	X	
	*Dicrostonyx groenlandicus*	Northern collared lemming	X	X
	*Glis glis*	European edible dormouse		X
	*Lasiopodomys brandtii*	Brandt's vole	X	X
	*Lemmus lemmus*	Norwegian lemming	X	X
	*Meriones unguiculatus*	Mongolian gerbil	X	X
	*Mesocricetus auratus*	Golden hamster		X
	*Microtus agrestis*	Short-tailed field vole		X
	*Microtus californicus*	California vole	X	
	*Microtus pennsylvanicus*	Eastern meadow vole	X	X
	*Microtus ochrogaster*	Prairie vole	X	X
	*Mus musculus domesticus*	House mouse (laboratory strains)	X	X
	*Mus musculus*	House mouse (wild)	X	X
	*Neotoma lepida*	Desert woodrat		X
	*Ondatra zibethicus*	Muskrat	?	?
	*Peromyscus californicus*	California deer mouse	X	X
	*Peromyscus leucopus*	White-footed mouse	X	X
	*Peromyscus maniculatus*	Eastern deer mouse	X	X
	*Phodopus campbelli*	Campbell's dwarf hamster	X	X
	*Phyllotis darwini*	Darwins's leaf-eared mouse	X	X
	*Rattus norvegicus*	Norway rat	X	X
Sciuridae	*Cynomys gunnisoni*	Gunnison prairie dog	X	X
	*Cynomys ludovicianus*	Black-tailed prairie dog	X	X
	*Cynomys parvidens*	Utah prairie dog	X	
	*Ictidomys tridecemlineatus*	Thirteen-lined ground squirrel	X	
	*Marmota caligata*	Hoary marmot	X	X
	*Marmota caudata*	Golden marmot	X	
	*Marmota flaviventris*	Yellow-bellied marmot	X	X
	*Marmota marmota*	Alpine marmot	X	
	*Otospermophilus beecheyi*	California ground squirrel		X
	*Paraxerus cepapi*	Smith's bush squirrel	X	
	*Urocitellus armatus*	Uinta ground squirrel	?	?
	*Urocitellus beldingi*	Belding's ground squirrel	X	X
	*Urocitellus columbianus*	Columbian ground squirrel	X	X
	*Urocitellus parryii*	Arctic ground squirrel	X	
	*Urocitellus richardsonii*	Richardson's ground squirrel		X
	*Urocitellus townsendii*	Townsend's ground squirrel	X	

Based on Ebensperger and Blumstein (2007), with the addition of the European hamster (Tissier et al., 2017), Azara's agouti (Smith and Smith, 2019) and the naked mole-rat (Wetzel et al., 2023). The question marks represent species in which infanticide is known, but the sex of the pup-killers is unknown.

Notably, rodents tend to show infanticidal behavior toward unrelated pups and parental behaviors toward their own pups. In most cases, pup-killing is selective and specifically targets alien (non-progeny) pups. Therefore, non-parental infanticide behaviorally dissociates from parental infanticide. Nevertheless, the inhibition of pup-killing induced by the parental state is so strong that often it not only prevents rodents from harming their own pups, but it also inhibits the killing of alien pups.

The mechanisms regulating male non-parental pup-killing were revealed by the ethologist Robert William Elwood through a series of seminal experiments beginning from the 1970s. Interestingly, Elwood discovered that male infanticidal behavior is inhibited by experience of the mated female's pregnancy. In particular, experiments with Mongolian gerbils exposed to alien pups showed a drastic inhibitory effect of the exposure to the mated female's pregnancy on the male's pup-killing (Elwood, 1977). This effect was confirmed by three rounds of experiments (round 1: Elwood, 1977; round 2: Elwood, 1980; round 3: Elwood and Ostermeyer, 1984) and Elwood then presented a pooling of the experiments (Elwood and Ostermeyer, 1984) showing results based on large sample sizes (153 subjects in total). The behavioral paradigm designed by Elwood featured males that were housed with females and exposed to pup tests in different periods. In these pup tests, the male's paired female was removed from the home-cage and the male was exposed to alien pups. Litter-naïve males (males that had never experienced rearing a litter) housed with a non-pregnant female, when exposed to the pup test, treated the pups as food and attempted cannibalism (which was interrupted by the experimenter) in over 60% of the cases (Elwood and Ostermeyer, 1984). However, after their paired female became pregnant, the pup-killing behavior of the males progressively diminished, decreasing to 30% in the period between 25 and 19 days pre-partum and falling down to about 10% in the period between 18 and 7 days before parturition. In the last week of the female's gestation (6–0 days pre-partum), the males completely ceased any attempt to kill the pups and never harmed any pup.

Remarkably, this inhibition of non-parental pup-killing can be long-lasting. Indeed, male gerbils that had previously generated a litter never harmed any pup in the pup test, regardless of the phase of the paired female's pregnancy (Elwood, 1977). Curiously, the infanticide inhibition is still reversible in male gerbils that have experienced only their mate's pregnancy, but have not yet had experience with the generated litter, while it is permanent in males that have previously reared a litter. Basically, if the pregnant female was removed from the litter-naïve male's cage, in tests performed immediately after (1 min later) the male did not kill alien pups, but if the female remained away for 1–3 days, then 50% of the males resumed pup-killing (Elwood, 1980). Conversely, in males that had had experience with their own litter, the removal of the female never reinstated pup-killing, even after 3 days of the female's absence (Elwood, 1980). This inhibition of pup-killing and the switch from male infanticidal behavior to paternal behavior has subsequently been confirmed in several rodent species, including laboratory house mice (Elwood, 1985), wild-caught house mice (McCarthy and vom Saal, 1986), Norway rats (Brown, 1986; Mennella and Moltz, 1988a), white-footed mice (Wolff and Cicirello, 1991), Eastern deer mice (Wolff and Cicirello, 1991) and California deer mice (*Peromyscus californicus*) (Gubernick et al., 1994).

Notably, as has been revealed in house mice (Palanza and Parmigiani, 1991) and Norway rats (Brown, 1986), the female-mediated inhibition of males' infanticidal behavior is not induced by mating *per se*, but by the subsequent cohabitation with the pregnant female. Mating *per se*, not followed by the cohabitation, led to little (Palanza and Parmigiani, 1991) or no (Brown, 1986) inhibition of male infanticide. Consistently, copulation followed by cohabitation with a non-pregnant female (ovariectomized in Brown, 1986; after induced abortion in Palanza and Parmigiani, 1991) did not inhibit male infanticide. Post-copulatory cohabitation with a pregnant female in a divided cage, where a wire mesh kept the male and the female physically separated, was sufficient to induce infanticide inhibition in virtually all males, highlighting the importance of female-released airborne olfactory signals (Palanza and Parmigiani, 1991). However, the mere cohabitation with a pregnant female without prior mating (i.e., cohabitation in the same cage but divided by wire mesh) failed to inhibit male infanticide (Palanza and Parmigiani, 1991). It therefore appears that, for infanticide inhibition, mating works as a priming factor, while the post-mating cohabitation with the pregnant female works as the actuating factor. Based on these results and on subsequent studies on the role of the vomeronasal system in infanticide (Mennella and Moltz, 1988b), it has been proposed that the female releases a low-volatility chemical signal, in particular a pheromone affecting the male's vomeronasal system, to prepare the male for paternity (Mennella and Moltz, 1988a,b; Elwood and Stolzenberg, 2020). Interestingly, even without any previous mating, cohabitation with a parturient female (impregnated by a different male) inhibited the male's infanticide in about 40–50% of the cases (Palanza and Parmigiani, 1991). It therefore seems that the female releases two chemical signals, one during pregnancy and one peripartum; the first signal inhibits infanticide conditional on prior mating, while the second has an unconditional inhibitory effect.

Females can exhibit non-parental infanticide even more than males. When tested in the paradigm described above for male gerbils (exposure to a pregnant female), litter-naïve female gerbils showed strikingly higher percentages of infanticide than males in all periods (Elwood, 1977). At >25 days pre-partum (when the stimulus female was non-pregnant), 100% of the subject females displayed infanticidal behavior. During the stimulus female's gestation, extremely high percentages of subject females continued to harm the pups (25–19 days pre-partum: 86%; 18–13 days: 100%; 12–7 days: 85%; 6–0 days: 50%). Moreover, unlike male gerbils, female gerbils did not show infanticide inhibition after litter-rearing experience (Elwood, 1977). Litter-experienced females harmed the pups in extremely high proportions across all periods (>25 days pre-partum: 100%; 25–19 days pre-partum: 100%; 18–13 days: 100%; 12–7 days: 100%; 6–0 days: 62%).

The capybara, the world's largest rodent, is a semiaquatic herbivore native to South America (Alvarez and Kravetz, 2006). Captive breeding of capybaras may be challenging, as under these conditions the percentage of non-parental infanticide is particularly high (Siqueira da Cunha Nogueira et al., 1999). Indeed, when primiparous females were housed in reproduction pens (in a group with multiple females), almost half of the pups (48%) were killed. In contrast, when primiparous females were housed in reproduction pens (isolated), 0% of the pups were killed. In nature, female capybaras spontaneously depart from the group some hours before giving birth, returning to the group with their offspring only several days after parturition (Siqueira da Cunha Nogueira et al., 1999). This is likely a naturally selected maternal countermeasure to avoid non-parental infanticide by the other females. In captivity, an analogous separation of the mother from the group prevents strikingly high percentages of non-parental infanticide. Interestingly, familiarity between the females in a reproduction group strongly affects infanticide (Siqueira da Cunha Nogueira et al., 1999). Comparison of reproduction pens with unfamiliar females and pens with familiarized females (which had lived together in the same pen at least since weaning) showed that in the first case 71% of the pups were killed, while in the previously familiarized groups 0% of pups were killed.

Female non-parental infanticide is also modulated by the subject's reproductive phase. For example, in eastern meadow voles (*Microtus pennsylvanicus*), killing of alien pups was frequent in pregnant females, less frequent in non-pregnant sexually mature females and sexually immature females, and virtually absent in lactating females (Ebensperger et al., 2000). In rats, virtually all nulliparous females exposed to alien pups killed them, while virtually no parous females (mothers) killed alien pups (Peters and Kristal, 1983). In mice, virgin females were typically indifferent or maternal toward alien pups, but even a brief food deprivation (just 3 h) was sufficient to bring the proportion of pup-attacking females to over 50% (Cao et al., 2025). Notwithstanding their natural tendency toward pup-killing, nulliparous females can be primed to motherhood. Indeed, when virgin female mice were exposed to either continuous cohabitation with a lactating dam and its litter (group 1: godmothers) or daily pup-habituation sessions (group 2: pup-sensitized females), both groups of females displayed maternal behaviors in the pup test (Martín-Sánchez et al., 2015).

Both males and females often eat the pups after killing them. Pup cannibalization has been observed in several rodent species, such as house mice, Norway rats, Mongolian gerbils, white-footed mice, eastern deer mice, eastern meadow voles, California ground squirrels, Belding's ground squirrels (*Urocitellus beldingi*), Townsend's ground squirrels (*Urocitellus townsendii*), thirteen-lined ground squirrels (*Ictidomys tridecemlineatus*), yellow-bellied marmots (*Marmota flaviventris*) and black-tailed prairie dogs (Ebensperger and Blumstein, 2007). Apparently, post-killing consumption would suggest that the function of non-parental infanticide is feeding. In this case, infanticide would be nothing but a predatory behavior. However, several facts suggest that this is not the case. First, post-killing cannibalism of pups can be absent in many species, including white-footed mice, eastern deer mice, eastern meadow voles, bank voles, short-tailed field voles (*Microtus agrestis*), Norwegian lemmings (*Lemmus lemmus*) and northern collared lemmings (Ebensperger and Blumstein, 2007). Second, pup cannibalism is observed even in rodent species whose diet is primarily herbivorous or occasionally insectivorous but not flesh-based, for example the yellow-bellied marmot. Third, feeding occurs multiple times per day almost every foraging day. In contrast, for a single individual, pup cannibalization may occur just a few times in a lifetime. Compared to the total number of meals in their entire lives, it is unlikely that evolution provided these rodents with a very specific pup-killing instinct just to obtain a few meals in a lifetime.

It appears that other functions sustain the existence of the pup-killing instinct in rodents. A likely possibility is reduction of sexual competition, i.e., reducing the chances of survival of conspecifics' offspring relative to those of one's own offspring. This explanation would be valid for both males and females. For females, non-parental infanticide could lead to acquisition of space and conservation of resources that could be used for their own offspring. On the other hand, for males, non-parental infanticide could additionally lead to acquisition of mates, since a lactating female is not fertile, but the destruction of the litter may bring the female back into estrus. Basically, for both females and males, non-parental pup-killing could be a strategy to increase the chances of survival of the individuals carrying copies of their alleles, at the expense of the conspecifics that do not carry them.

Particularly useful insights regarding the sexual competition hypothesis could come from the observation of rodent societies where the inter-individual disparity in the reproductive chances is extreme, like in naked mole-rat colonies, which are divided into a breeding and a non-breeding caste. Interestingly, in this species, pup survival rate has been found to be negatively associated with the number of non-breeding individuals (Wetzel et al., 2023). Pups raised by the breeding pairs alone had a survival rate of 90%. In contrast, for pups in colonies of more than 37 individuals, the survival rate dropped to less than 50%. Moreover, members of the non-breeding caste have been seen killing and eating the newborns of the breeding caste (Wetzel et al., 2023). It is unknown why non-breeders behave like this. However, enough is known about the context of this behavior to allow the formulation of hypotheses. More specifically, this behavior was performed in conditions where the animals had *ad libitum* access to food. When a breeder dies, the individuals from the non-breeding caste have the chance to replace it. Consequently, killing other non-breeders enhances the killer's chances of eventually becoming a breeder and producing offspring of its own. All newborn naked mole-rats automatically belong to the non-breeding caste (and newborns are easier to kill than adults). Altogether, these facts suggest that pup-killing by non-breeding naked mole-rats is more likely related to sexual competition rather than an attempt to obtain food. Additionally, it has been proposed that pup-killing by non-breeders could be a form of selection of the fittest pups, which would benefit the colony as a whole (Wetzel et al., 2023). While further research is necessary, naked mole-rat colonies, with their clear division between parents and non-parents, and with their highly asymmetric numbers of parents and non-parents, could be a promising model to better investigate the nature of non-parental infanticide.

### 5.2 Parental infanticide

In some cases, rodents may kill their own pups. This behavior primarily concerns very young offspring and typically occurs shortly after parturition. Parental infanticide is well-known in laboratories hosting breeding colonies of rodents. In such breeding colonies, pups are counted daily and, with a certain frequency, pups are found dead or missing. This perinatal mortality is commonly monitored, as it is an important factor limiting breeding success. When pups are missing, it means that the bodies of the pups have been cannibalized by an adult, and generally it is assumed that cannibalized pups have been killed. Remarkably, the standard daily monitoring may miss the pups that are cannibalized on the first day, before they can be recorded (i.e., in the hours between birth and the first daily check). These pups are not even recorded as born, so they surely cannot be counted as deaths. Consequently, the number of pup deaths can easily be underestimated. A recent study addressed this issue by comparing the classical daily cage inspection method with continuous video monitoring (Brajon et al., 2021). This study found that perinatal pup death is indeed underestimated by conventional monitoring. Compared to the daily inspection method, video monitoring reported litter sizes that were 35% greater and pup death tolls that were 102% higher.

Regarding infanticide, an important point is that, while dead or missing pups have long been considered to be the result of killings, with the standard monitoring methods infanticides are not directly observed. For instance, in the definition of Garner and colleagues, infanticide includes not only the cases in which animals are observed killing pups, but also “litters born and disappearing” and “finding parts of pups” (Garner et al., 2016). Interestingly, the aforementioned study by Brajon et al. (2021) additionally performed an analysis of infanticidal behavior, finding that it is actually less common than expected. When females were housed alone with their pups, it was found that 171 of 472 (36.2%) born pups died within postnatal day 4. Of the dead pups, 21.6% were cannibalized. Whenever possible (in all cases where the pup was visible in video before the act of cannibalization), it was determined whether cannibalized pup had been killed or not, and the statistics were calculated. Remarkably, it was found that 0% of the cannibalized pups had been killed by the mother. In cages where the dam mouse was housed in a trio (with the litter's father and another female), infanticide was present, but it was still uncommon. Pup-killing had occurred only for less than 25% of the cannibalized pups, indicating that in the vast majority of the cases (over 75%) the cause of the pup's death was not infanticide. The infanticides were always performed by the females. In the trio cages, infanticides were caused by the dam in 30–40% of the cases and by the cagemate female in 60–70%. Notably, paternal infanticide was 0%. The males initiated extremely few (less than 4%) acts of cannibalism and never killed a pup (when they cannibalized, they exclusively ate pups that were already dead). After an adult had initiated an act of cannibalism, other adults could join the first animal. The participation of the females in the cannibalistic events was almost total (99.2% for the cagemate female and 98.4% for the dam), while the males participated only in 48.8% of the cases. However, of the total number of pup deaths, the cases of infanticide were 0% in the solo mother cages and below 9% in the trio cages, highlighting a very low contribution of infanticide to perinatal death. Consistently, another study, which performed video analysis of single-housed female mice that had lost their entire offspring before weaning, did not find a single case of infanticide (Weber et al., 2013). Hence, while it is an existing phenomenon, parental infanticide is likely less common than had been thought until recently.

The findings described above suggest that early pup mortality is largely driven by non-infanticidal factors, including parturition-related complications, thermoregulatory failure, failure to initiate effective suckling, or early developmental defects, with cannibalism often occurring secondarily. In support of this idea, a recent investigation performed necropsies on 324 dead mouse pups to determine the cause of death (Capas-Peneda et al., 2020, 2024). Strikingly, 22% of the dead pups were found to be stillborns, as revealed by analyses of lung morphology, lung float test, stomach content and brown adipose tissue coloration. Furthermore, among the remaining dead pups, 97% died because of inability to initiate successful suckling (only 3% of the liveborns had milk in their stomach). External traumatic lesions were found in 25% of the livebirths and 7% were partially eaten. However, these lesions could have been caused post-mortem, since 19% of the stillborns showed traumatic lesions as well. Internal bleeding, compatible with live traumatic damage, was found in only 4% of the total number of deaths (6% of the livebirths). Overall, starvation was the leading cause of death of the pups, followed by stillbirth.

Remarkably, ecological factors such as food-type availability (and consequent diet composition) can dramatically affect parental infanticide. For example, intensive maize monocultures can strongly increase pup-killing in female European hamsters (*Cricetus cricetus*). Maize is a cereal known for its lack of bioavailable vitamin B (Nuss and Tanumihardjo, 2010). After the importation of maize from the New World, a dietary revolution occurred in Europe (Mariani-Costantini and Mariani-Costantini, 2007). However, as a consequence of quasi-monophagic maize diets, especially in the socioeconomically disadvantaged groups, an epidemic of pellagra (a disease caused by severe vitamin B3 deficiency) affected millions of people and, between 1735 and 1940 alone, killed hundreds of thousands of people in Europe and North America (Mariani-Costantini and Mariani-Costantini, 2007; Bryan and Mull, 2015; Viljoen et al., 2018; Jaworek et al., 2021). In the laboratory, it has been observed that female European hamsters fed maize-based diets failed to show maternal behaviors (Tissier et al., 2017). They did not give birth within the nest and they did not retrieve the newborn pups to carry them to the nest. Rather, the dams left the pups scattered across the home-cage. Subsequently, the females started placing the pups on top of their hoard of maize grains, and then ate them. In the wheat-worm diet group, the rate of pup survival from birth to 30 days (weaning) was about 80%. In contrast, in the maize-worm diet group pup survival dramatically dropped to less than 5%. Among the surviving pups, cases of siblicide were also observed. In particular, at 34 days of age, two males cannibalized their female siblings, eating them while they were still alive. Notably, these two diets had similar macronutrient contents (wheat-worm diet: 42% proteins, 43% carbohydrates and 15% lipids; maize-worm diet: 46% proteins, 40% carbohydrates and 14% lipids), ruling out the possibility that pup-killing was induced by a macronutrient deficiency. Instead, it was found that the infanticidal behavior of the female hamsters was due to micronutrient deficiency. Indeed, diet supplementation with vitamin B3 suppressed infanticide and restored functional maternal behaviors in the female hamsters, leading to an 85% increase in the number of weaned pups in the maize-worm-B3 diet group compared to the maize-worm diet group (Tissier et al., 2017).

Parental infanticidal behavior is generally positively modulated by environmental stressors (Kristal, 2009), such as overcrowding (Calhoun, 1962), noise (Poley, 1974; Jafari et al., 2017), cold temperature (Zafar et al., 2018), vitamin deficiency (Bâ, 2013; Tissier et al., 2017) and food scarcity (Schneider and Wade, 1989; König, 1989). In laboratory breeding facilities, where temperature and food availability are constant and favorable, parental infanticide is likely a maladaptive behavior rather than an evolved parental strategy. Nevertheless, maternal infanticide can in principle be adaptive under exceptional circumstances, because gestation and especially lactation represent substantial energetic investments, and terminating investment in non-viable or low-prospect offspring may allow a faster return to reproduction under improved conditions. In laboratory settings, maternal infanticide is a recurrent phenomenon, likely due to artificial housing conditions that impose chronic stress and disrupt normal parental behavior, although its prevalence has probably been overestimated because pup disappearance and cannibalism have often been equated with active killing. In wild rodents, by contrast, stable territories, much lower encounter rates with conspecifics, and concealed nesting sites are expected to impose strong selective pressure against parental infanticide, as killing one's own offspring would directly compromise reproductive success, and accordingly there are very few reports of true parental infanticide in free-ranging rodent populations. In sum, parental infanticide in rodents appears to be a relatively rare and context-dependent behavior that may be adaptive only under exceptional circumstances, such as when offspring survival is severely compromised and continued parental investment would reduce future reproductive success (Hrdy, 1979).

## 6 Ethical issues related to research on rodent killing behaviors and future directions

From the 18th century to the present there has been an increasing recognition of the importance of the welfare of the animals with which humans interact (for a brief history of the rise of stances advocating animal welfare, see section 5.1 of d'Isa and Abramson, 2025). In biomedical research, the number of papers mentioning “animal welfare” has exploded from the 1980s to the present day (d'Isa et al., 2024b). Research on predation, intraspecies adulticide and infanticide commonly features the killing of animal subjects. This poses a significant ethical issue. Robert Elwood was the first, over 30 years ago, to devote an entire paper to this critical issue (Elwood, 1991). For many behavioral domains, in accordance with the 3Rs (Replacement, Reduction and Refinement) principles, it has been possible to develop refined behavioral tests that do not cause death, harm or pain to the involved animals. For domains like memory, researchers have been able to design animal-friendly tests avoiding painful or stressful motivators, such as electric shocks or prior food deprivation. Examples are the object recognition test for recognition memory (d'Isa et al., 2014) and the hole-board test for spatial memory (d'Isa et al., 2021). Unfortunately, in research on killing behaviors, it is more difficult to develop refined alternatives, as the killing behavior is the outcome variable itself. Nevertheless, several options are conceivable to address this ethical issue.

First, following the replacement principle, in predatory killing tests it would be possible to employ, as stimulus animals, invertebrates (e.g., insects) rather than vertebrates. For instance, when testing rat predation, the researchers could decide to use the cricket-killing test instead of the mouse-killing test. Insect-killing and rodent-killing may not share exactly the same neural underpinnings. For example, in grasshopper mice it has been seen that the anti-aggressive drug fluprazine inhibits the killing of house mice, but not of crickets (Schultz and Kemble, 1986). This may be due to the fact that mouse-killing incorporates elements of intraspecies aggression. Analogously, while rat mouse-killing is predatory (O'Boyle, 1974, 1975; Rossi, 1975), it also incorporates some aspects of intraspecific aggressiveness (Barr et al., 1975; Van Hemel, 1975). Consequently, for both rats and grasshopper mice, the choice of using cricket-killing as a model of predation would not only optimize animal welfare, but also provide a purer and more ethologically valid measurement of predation (Kemble et al., 1985).

Second, it may be possible to adopt the method of interrupting killing behaviors upon their initiation. This approach could allow successful scoring of the killing behaviors of the subject animal while limiting the harm to the stimulus animal. For instance, when studying infanticide, Elwood decided to stop the adult's attacks at the first bite of the pup, and he managed to obtain very informative and solid results, confirmed by several experimental replications (Elwood, 1977, 1980; Elwood and Ostermeyer, 1984). Similarly, the team of Jaap Koolhaas proposed a protocol for testing potentially lethal aggression in which this type of attack is interrupted at its commencement (Koolhaas et al., 2013). In mice, intraspecies attacks can be divided into functional aggression and violence, which is a pathological and injurious form of offensive aggression that is out of control and out of context (Koolhaas et al., 2013). Violence is not subject to inhibitory control and is dysfunctional, as it does not have any additional functional value compared to regular aggression and it may even be directed at inappropriate subjects (such as a potential mate). Notably, violence differs from aggression not just quantitatively, but also qualitatively. Indeed, in regular aggression the most vulnerable body parts are off-limits and spared from attack. Attacks are concentrated mostly on areas with thick skin such as the back and the flanks (Miczek et al., 2001). In contrast, violence features bites to extremely vulnerable body parts such as the throat, the abdomen and the paws (Haller and Kruk, 2006; Koolhaas et al., 2013), which makes the attacks potentially lethal. As the targets of lethal attacking are different from those of regular aggression, violence can immediately be recognized at its initiation and it is not necessary to let the subject animal kill the stimulus animal. Upon its commencement, such attacking behavior can easily be scored as violent and the fight can be stopped at that point by the experimenter. An alternative option, similarly based on the scoring of behavior initiation, is to provide protection to the stimulus animals. For example, pups have been presented to potentially infanticidal adults in wire mesh tubes that allow the tested adult to sniff and attack the enclosed stimulus pup, while preventing the pup from being injured (Korpela et al., 2010).

Third, considering the reduction principle, stimulus animals could be substituted by dummies or robots (d'Isa, 2025a). In the case of infanticide testing, the stimulus animal is a pup. Rubber dummies, being static, may elicit low or no pup-attacking. Multimodal stimulation would be preferable, and robotic pups could provide this option. As pups display very elementary movements, it could be easy to design soft robots reproducing both their appearance and their motor behavior. Moreover, as live pups produce ultrasonic vocalizations that play a role in modulating adult-infant interactions (Branchi et al., 2001), the robotic pups could be endowed with the ability to emit ultrasonic stimuli pre-recorded from live pups. Finally, when using such robotic pups, an important aspect would be to provide the robots with the appropriate olfactory cues, for example collected from real pups. Indeed, it has been shown that pup odors activate the vomeronasal organ (VNO) of adult males, inducing pup-attacking (Elwood and Stolzenberg, 2020). Lesion of the VNO inhibits infanticide in both male mice and male rats (Izquierdo et al., 1992; Mennella and Moltz, 1988b; Tachikawa et al., 2013), and functional silencing of VNO neurons in male mice produces the same effect (Wu et al., 2014; Trouillet et al., 2019). Robotic pups bearing different odors could be employed to further explore the role of the VNO in mediating pup-killing. In addition to infanticide, animal robots could be useful to study predation as well. Robotic crickets or cockroaches could be used to study rodent predatory behavior (d'Isa, 2025a). Cockroach-like robots have recently been employed with mice, and they were successful in eliciting predatory behavior (Rossier et al., 2021). Intriguingly, robotic crickets could be remotely controlled, enabling the experimenters to make them hop at will, eliciting predatory chasing by the rodents at specific moments (and in specific directions), as most appropriate for the chosen experimental design.

Finally, another approach could be to study the killing behaviors of free-living rodents. In this case, the killings take place anyway in nature, independently of the researchers' study. However, as killing behaviors are isolated and often unpredictable, it is very difficult to observe the exact moment when a killing act occurs. A solution to this issue is to employ methods based on long-term behavioral monitoring, such as motion-triggered video recordings covering periods of weeks or months. Over the course of the past few years, our team has used a method of this type to observe thermoregulatory behaviors (Stryjek et al., 2021, 2024), intraspecific agonistic behaviors (d'Isa et al., 2024a) and interspecific anti-agonistic behaviors (deceptive dodging: d'Isa et al., 2024a; temporal niche switching: Stryjek et al., 2026). Very recently, through this same method, we obtained the first video documentation of an *Apodemus* mouse killing a heterospecific rodent, more specifically a lethal attack by the yellow-necked mouse upon the bank vole (Kurek et al., 2026). Additionally, with the constantly progressing miniaturization of video cameras, animal-borne cameras placed on free-living individuals, an approach which has already been carried out with rodents (La Londe et al., 2015; Poling, 2016; d'Isa, 2025d), could in the future provide further insights into rodents' lethal actions in nature.

Intriguingly, much remains to be discovered regarding the targets, the contexts and the forms of rodent killing behaviors. A more careful observation of rodent killing behaviors in nature may contribute to shedding more light on the natural instincts of species that are currently the most widely employed in biomedical research. Moreover, a better understanding of the contexts triggering the killing behaviors makes it possible to avoid the killings when such an outcome is desired, for instance in laboratory breeding colonies. Furthermore, an investigation of the neural substrates of killing, together with an analysis of the individual differences between killers and non-killers, could lead to a better understanding of the ultimate nature of killing tendencies, and some of the resulting findings could have such broad cross-mammalian generalizability that they would shed light on the nature of human killing behavior as well (Blanchard and Blanchard, 2003; Nelson and Trainor, 2007; de Boer, 2018; Potegal and Elwood, 2026). Finally, rodent models of reduction of killing behaviors and violence could be developed, for example through environmental and pharmacological interventions, and some of the findings could potentially be translated to humans. These insights could inform the development of social preventive strategies and specific educational programs. Furthermore, they could contribute to designing targeted, consensual pharmacological treatments aimed at regulating specific neurochemical dysfunctions associated with pathological aggressiveness.

## Footnotes

1 Translated from French by Raffaele d'Isa. Original French text: “pour la plupart herbivores ou fructivores, quelquefois omnivores” (Gervais, 1854).

2 Director of the Natural History Museum of London.

3 We define a killing instinct as an innate lethally attacking behavior that is unconditionally triggered by the detection of specific features of a potential target. Instinctual killing behaviors are stimulus-specific, highly stereotyped, largely shared by the individuals of a species and hereditable.

4 Also known as striped field mouse.

5 Also known as Siberian hamster, striped hairy-footed hamster and winter white dwarf hamster.

6 Analysis of the stomach content of about 300 individuals of this species found that animal matter averaged at 73.8%, peaking at 85.6% in the late spring/early summer period (Flake, 1973).

7 For instance, in his zoological encyclopedia, the Swiss naturalist Conrad Gessner (1516-1565), after devoting a section to the house mouse (entitled *De Mure*), presented a section devoted to other types of mice (*De Muribus Diversis*), in which he included the shrew (described in the subsection *De Mure Araneo*) (Gessner, 1551). The graphical representation provided by Gessner for the common shrew (which he referred to as *Mus araneus*, instead of *Sorex araneus* as it is currently defined) is reproduced in Supplementary Figure 1. Analogously, the Italian naturalist Ulisse Aldrovandi (1522-1605), the first professor of natural history at the University of Bologna, presented shrews as *Mus araneus* and showed comparative anatomical figures to compare the upper jaws, lower jaws and teeth of shrews and house mice, which he referred to as *Mus domesticus* (Aldrovandi, 1637).

8 Previously known as *Chibchanomys orcesi*, until it was moved into the *Neusticomys* genus in 2023 (Salazar-Bravo et al., 2023).

9 “Avenue of the Volcanoes”, episode 5 of season 13 of the British documentary series “Natural World”, broadcast on 22 January 1995 (https://www.imdb.com/title/tt1745363/).

10 Cardano was professor of medicine at the University of Pavia from 1543 to 1551. In his 1550 work, he writes that he saw the animal in January of the “current year”, so he likely observed the porcupine in January 1550 or 1549.

11 In the 1540s, Mattioli had published an Italian translation and commentary of *De materia medica* by Pedanius Dioscorides (c. 40-90 AD), a Greek physician and herbalist who lived in Rome under the Neronian Empire (Mattioli and Dioscorides, 1544). In the seventh Italian edition (Mattioli and Dioscorides, 1555) and in the first Latin edition (Mattioli and Dioscorides, 1554) of this commentary, Mattioli added a brief section on the porcupine (a species which was absent in the previous editions), as well as an image comparing the appearance of the porcupine and the hedgehog. Three years before Mattioli, Gessner had also provided a graphical representation of the porcupine (Gessner, 1551). However, Mattioli's figure is anatomically more accurate. The porcupine representations provided by Gessner and Mattioli are shown in Supplementary Figure 2.

12 “Agonistic behavior”, a phrase coined by the comparative psychologists John Paul Scott (1909-2000) and Mary-‘Vesta Marston (1924-2024) in 1950 (Scott and Marston, 1950; d'Isa, 2025c), includes, by definition (Scott and Fredericson, 1951; Scott, 1966), not just actual attacks but also pre-attack signaling of attacking capacity and readiness.

13 In current laboratory assessments, in order to minimize the probability of fatal injuries, the standard duration of the resident-intruder test is generally limited to 10 minutes (Koolhaas et al., 2013).

14 Translated from French into English by Raffaele d'Isa.

15 Previously thought to be a subspecies of *Peromyscus maniculatus*, it was known as *Peromyscus maniculatus gambeli* until 2017, when molecular phylogenetic analysis led to a reclassification (Greenbaum et al., 2017).
